# Monte Carlo simulation of cylinders with short-range attractions

**DOI:** 10.1063/1.5040252

**Published:** 2018-09-12

**Authors:** Harold W. Hatch, Nathan A. Mahynski, Ryan P. Murphy, Marco A. Blanco, Vincent K. Shen

**Affiliations:** 1 Chemical Informatics Research Group, Chemical Sciences Division, National Institute of Standards and Technology, Gaithersburg, Maryland 20899-8380, USA; 2 Center for Neutron Science and Department of Chemical and Biomolecular Engineering, University of Delaware, Newark, Delaware 19716, USA; 3 Institute for Bioscience and Biotechnology Research, University of Maryland, Rockville, Maryland 20850, USA

## Abstract

Cylindrical or rod-like particles are promising materials for the applications of fillers in nanocomposite materials and additives to control rheological properties of colloidal suspensions. Recent advances in particle synthesis allows for cylinders to be manufactured with short-ranged attractions to study the gelation as a function of packing fraction, aspect ratio and attraction strength. In order to aid in the analysis of small-angle scattering experiments of rod-like particles, computer simulation methods were used to model these particles with specialized Monte Carlo algorithms and tabular superquadric potentials. The attractive interaction between neighboring rods increases with the amount of locally-accessible surface area, thus leading to patchy-like interactions. We characterize the clustering and percolation of cylinders as the attractive interaction increases from the homogenous fluid at relatively low attraction strength, for a variety of aspect ratios and packing fractions. Comparisons with the experimental scattering results are also presented, which are in agreement.

## I. INTRODUCTION

Anisotropic shapes are ubiquitous in nature, often conferring unique adaptations over more symmetric counterparts. They manifest on a variety of length scales, which range from the internal structure of the atom, to the domains within proteins, to the internal organization of cells, and beyond. Anisotropy has become well appreciated in the field of colloidal science^1,2^ for its control over the material properties of suspensions.^3–7^ By tuning the particle shape and taking advantage of anisotropy, the self-assembly of colloidal particles can be carefully controlled.

Perhaps the simplest perturbation conferring anisotropy onto an otherwise isotropic object, is the elongation of a sphere into a spherocylinder. These rod-like particles occur in many materials such as cements, paints, pharmaceuticals and consumer products. They have been shown to be facile building blocks in the assembly of nanoscale polyhedrons,^8,9^ and are also observed in viruses.^10,11^ Historically, the study of athermal, hard rods has been the subject of great interest because the anisotropic shape of the rods can lead to orientational ordering transitions on the basis of entropy alone due to its transfer between rotational and translational modes.^12–14^ As the aspect ratio of the rods increases, their modes progressively become decoupled, which facilitates the formation of a variety of morphologies.^15^ For instance, interactions between grafted ligands can introduce greater complexity, and even be used to confer directional interactions.^7^ The addition of small polymers or other colloids to suspensions of rods can alternatively introduce depletion effects, which are also entropic in nature.^16,17^

The latter instance has been the source of much theoretical interest,^17–26^ as depletion effects are estimated to play a significant role in conditions where rods occur naturally, as in cellular environments,^27^ and in the manufacturing of nanocomposites^28^ and liquid crystals.^1,29,30^ Furthermore, understanding depletion effects may act as a surrogate for a plethora of other short-ranged interactions, regardless of their cause.^31–34^ These short-range attractions may lead to percolation which has implications for dynamical arrest^35–39^ and electrical conductivity.^40,41^ Although the connection between rigidity percolation, coordination number and gelation has been studied for isotropic particles,^42^ the phase diagram for rod-like particles^32^ possesses an additional degree of freedom to explore relative to spherical particles (i.e., the aspect ratio). The focus of this work is to study clustering and percolation over a variety of attraction strengths, volume fractions and aspect ratios.

Owing to the symmetry of the spherical caps at their termini, it is generally simpler to both simulate and synthesize colloidal spherocylinders rather than cylinders. Recent advances in experimental synthesis, however, have led to the ability to produce silica-based colloidal rods that have one spherical end while the other is flat.^43,44^ Although the termini of a cylinder are both flat, this intermediate structure reflects the growing necessity for theoretical and simulation methods that can be used to study the behavior of these systems.

To this end, we performed computer simulations of cylindrical particles with flat-ends and short-ranged attractions. We achieved efficient simulation of flat-end cylinders (not spherocylinders) by developing a numerical scheme to compute the interaction between convex superquadric solids of revolution. The surfaces of superquadric solids may be described analytically, which has been exploited in the past to study a subset of convex superquadrics known as superballs that can smoothly interpolate from a cube to a sphere and an octahedron.^45–47^ The addition of short-ranged attractions due to depletion has also been investigated recently.^48^ Superquadric shapes also have been simulated as hard rigid bodies^49^ and with attractive patches.^50^ Similarly, analytical solutions for the interaction potential between convex superquadric shapes and a planar wall under the influence of depletion have also been investigated.^51^

In this computational work, we study cylinders with an attraction range that is approximately 4% of the cylinder diameter, which have also been the focus of recent experimental studies.^43,52^ Thus, these systems represent an opportunity to compare experiment and simulation. It is also worth noting for systems with such short interaction ranges that the liquid state is expected to be metastable with respect to the solid.^53^ In addition, this study is limited to packing fractions of 22.5% or less, which is below the nematic and smectic regions of the phase diagram of hard spherocylinders. In this work, we study the clustering and percolation as a function of aspect ratio, packing fraction and attraction strength.

Although analytical methods have been developed to simulate hard cylinders,^54,55^ including those with patchy interactions requiring iterative procedures,^50,56^ we use a tabulated potential to speed up the simulation of superquadrics with attractions due to the required calculation of excluded volume overlaps between pairs of cylinders. This excluded volume overlap is the most computationally intensive contribution to the interaction potential. The tabular potential allows the interactions to be computed only one time for a given relative orientation and position before the simulations begin. With this procedure, the expensive calculation of the interaction between pairs of anisotropic particles is replaced by a query of the stored table. The relative efficiency of the tabular potential with respect to analytical pair wise calculations depends upon the specific model and the resolution of the table. Here, we first validate the simulations using the tabulated potential by calculating virial coefficients and scattering profiles. The virial coefficients by themselves yield a cross over from end-end dominant attractions to side-side dominant attractions as a function aspect ratio, and scattering profiles are compared with experiment. We then use the simulations to study metrics which signal percolation and dynamic arrest (e.g., cluster percolation probability, coordination number and orientational order), by simulating colloidal cylinders over a wide range of aspect ratios, attraction strengths, and packing fractions.

The outline of this paper is as follows. In Section II, we describe the model for the cylindrical particles and the interaction potential. To explore the effects of aspect ratio on the interaction potential, virial coefficient calculations are presented in Section III. The Monte Carlo methods, including Wang-Landau sampling and expanded ensemble approaches, used to simulate fluids composed of cylindrical particles are described in Section IV. In Section V, we compare simulated and measured scattering profiles to further test the model and simulation method. We then compare various metrics used to identify clustering and percolation in Section VI. Finally, conclusions are made in Section VII and the tabular potential for the computational modeling approach is detailed in the Appendixes.

## II. MODELS

**FIG. 1.**
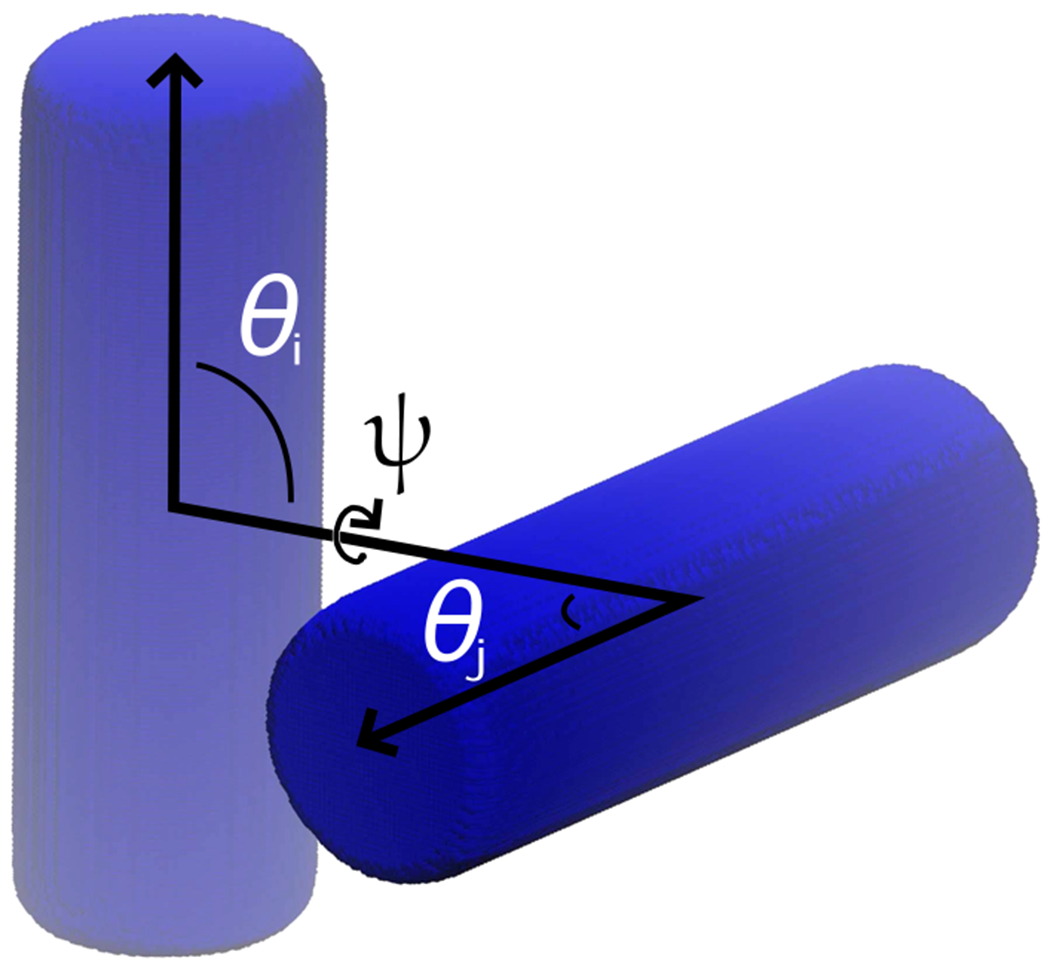
A pair of cylinders with *L/D* = 3, *ϵ*_1_ = 0.1, *θ_i_* = *θ_j_* = *ψ* = *π*/2 and *r* = 2*D*.

Cylindrical particles were modeled with the superquadric equation,^57^

(1) $${\left[{\left(\frac{x}{a_{x}}\right)}^{\frac{2}{\epsilon_{2}}}+{\left(\frac{y}{a_{y}}\right)}^{\frac{2}{\epsilon_{2}}}\right]}^{\frac{\epsilon_{2}}{\epsilon_{1}}}+{\left(\frac{z}{a_{z}}\right)}^{\frac{2}{\epsilon_{1}}}=1,$$

where *x, y* and *z* are the Cartesian coordinates, *ϵ*_1_ and *ϵ*_2_ parameters determine the curvature, and the *a_i_* parameters, with *i* = *x, y, z*, determine the maximum extent of the shape from the center, in the given dimension. To model cylinders, the constraints *a_x_* = *a_y_*, *ϵ*_2_ = 1 are imposed, such that the length of the cylinder, *L* = 2*a*_*z*_, which extends in the *z* direction in the body-fixed frame of reference, and the diameter of the cylinder, *D* = 2*a_x_*. The parameter *ϵ*_1_ = 0.1 was chosen such that the ends of the cylinders were flat. An example is shown in Figure 1. As shown in the figure, the relative position and orientation of two solids of revolution, *i* and *j*, are given by the center separation distance, *r*, and three angles, *θ_i_, θ_j_*, and *ψ*. The first two angles, *θ*_*i*_ and *θ_j_*, are the angles formed between the center separation vector and the axis of revolution of a particles *i* and *j*. The third angle, *ψ*, is the dihedral angle formed by the three vectors described above (i.e., *ψ* is the angle between two planes formed by the center separation vector and the axes of revolution of each particle). The potential energy, *U*, between a pair of cylindrical particles, *i* and *j*, is *U* = *U*^*h*^ + *U*^*a*^ + *U*^*e*^, which includes a hard-particle steric repulsion, *U*^*h*^, a short-range attractive interaction, *U*^*a*^, and a screened electrostatic repulsion, *U^e^*. These contributions are described below.

The hard-particle interaction between two particles, *U*^*h*^, is given by

(2) $$U^{h}\left(r,\theta_{i},\theta_{j},\psi \right)=\{\begin{array}{cc} \infty & r<r_{h}\left(r,\theta_{i},\theta_{j},\psi \right)\\ 0 & r\geq r_{h}\left(r,\theta_{i},\theta_{j},\psi \right). \end{array}$$

where *r*_*h*_(*θ_i_, θ_j_, ψ*) is the hard center separation distance at contact, which is computed numerically using Equation (1) as described in Appendix B.

The short-ranged attractive interaction was modeled with the pair-wise implicit-depletant potential,

(3) $$\frac{U^{a}\left(r,\theta_{i},\theta_{j},\psi \right)}{\epsilon }=-\frac{\Delta V_{ex}\left(r,\theta_{i},\theta_{j},\psi \right)}{\Delta V_{ex}^{m}}$$

where Δ*V*_*ex*_ is the excluded volume overlap (for a hard sphere of radius *R*_*g*_ = 0.04D) between two cylinders, *i* and *j*, and $\Delta V_{\mathrm{ex}}^{m}$ is the maximum of Δ*V*_*ex*_ over all non-overlapping positions and orientations between a pair of rods. The parameter *ϵ* is the scale of the interaction strength. Note that the potential goes to zero at the interaction center separation cut off distance, *r*_*c*_, when the edges of the excluded volumes of the particles touch. A physical interpretation of Equation (3) may be obtained by considering the Asakura-Oosawa depletion force^16^ induced by a dilute solution of particles with a packing fraction of *ϕ_d_*. The potential energy is given by $U^{a}=-\frac{\Delta V_{\mathrm{ex}}(r,\theta_{i},\theta_{j},\psi )}{\frac{4}{3}\pi R_{g}^{3}}\phi_{d}k_{B}T$, where *k*_*B*_ is the Boltzmann constant and *T* is the temperature. Equation (3) is then obtained when *ϵ* is equal to the maximum of the absolute value of *U*^*a*^. The algorithm used to compute Δ*V_ex_*(*r, θ_i_, θ_j_, ψ*) is described in Appendix C. The excluded volume of a particle was approximated by Equation (1) with the size parameters *a_i_* → *a_i_* + *R*_*g*_, which is accurate for *a*_*x*_ ⪢ *R*_*g*_. For *a*_*x*_ ⪢ *R*_*g*_, the implicit depletant potential is more computationally efficient than explicitly simulating the depletant molecules, although there are alternative methods.^58^ With this implicit depletion treatment, many-body effects can arise when the excluded volume of more than two particles overlap,^59^ but these effects are expected to be small for *a*_*x*_ ⪢ *R_g_*.^48^ This model is amenable to studying systems with attractions that are physically distinct from the depletant interaction, but are also similarly short-ranged. For example, van der Waals attractions in colloidal systems (e.g., recently synthesized cylindrical particles^43^) may be accurately modeled with this short ranged potential by applying extended corresponding states.^60,61^

Finally, we include an electrostatic repulsive term, *U*^*e*^, which we assume to have a Yukawa form given by

(4) $$\frac{U^{e}\left(r,\theta_{i},\theta_{j},\psi \right)}{\epsilon }=\frac{D}{r}e^{-\kappa \left[r-r_{h}\left(\theta_{i},\theta_{j},\psi \right)\right]},$$

where the parameter *κD* = 10^4^ was chosen such that *U*^*e*^ only contributes over an extremely short distance of approximately 5*D* × 10^−4^. Note that this is an approximation of the electrostatic interaction, where a more rigorous treatment might require accounting for the Gaussian curvature by using the Derjaguin approximation.^51^ We note that simulations were also performed without Eq. (4) and we did not see a difference in the computed properties. This term is relatively insignificant for the results of this study due to the size of the parameter *κD* = 10^4^, relative to the strength of the attractive interactions, which we will discuss quantitatively in the following paragraph. But we used this term to make the model more amenable to molecular dynamics simulations with continuous potentials.

**FIG. 2.**
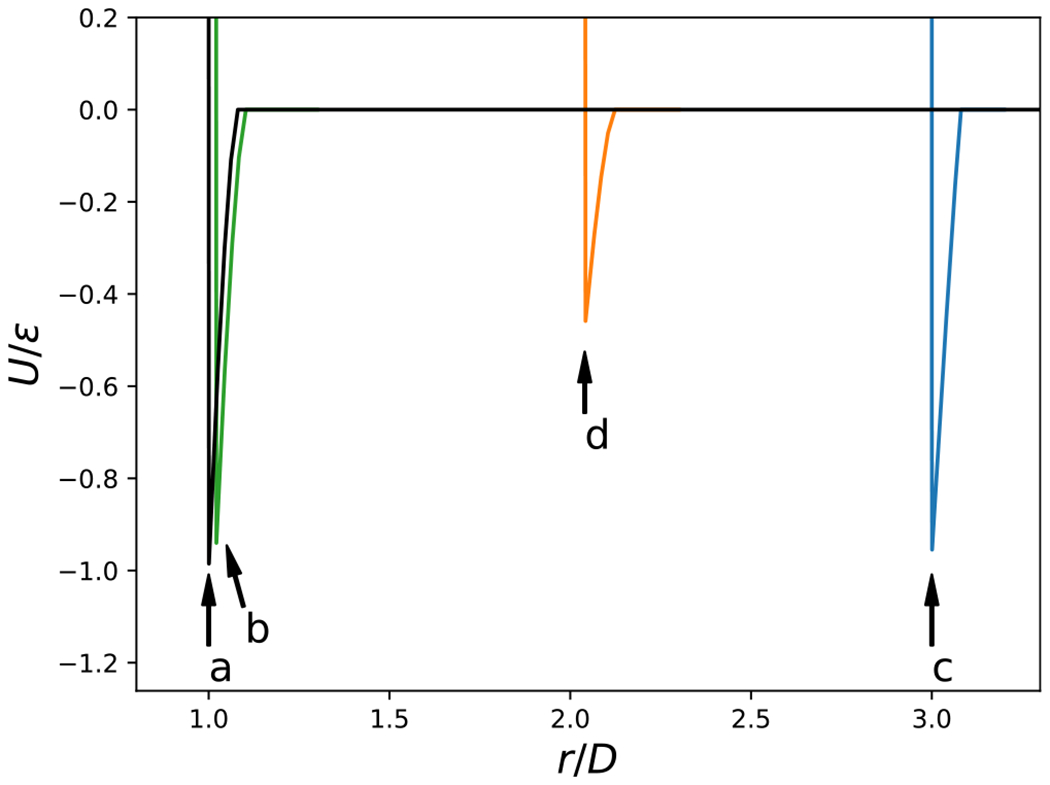
The total potential energy between a pair of cylinders with aspect ratio of *L/D* = 3 and short-ranged attractions, *R_g_/D* = 0.04, as a function of the separation distance of the centers for a few orientations. Letter-coded labels refer to structures shown in Figure 3.

**FIG. 3.**
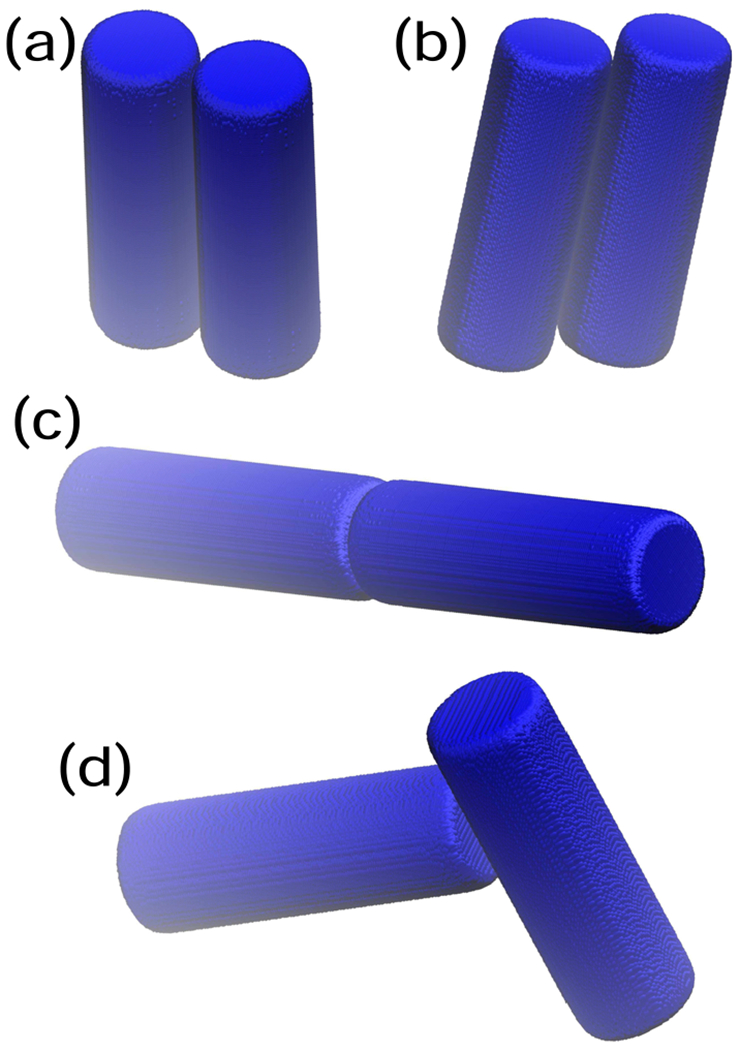
Example configurations of pairs of cylinders with aspect ratio of *L/D* = 3 and the following relative positions: (a) cos *θ_i_* = cos *θ_j_* = 0, and cos *ψ* = ±1, resulting in *r_h_* = *D* and *U^a^/ϵ* = −1 (b) cos *θ_i_* = cos *θ_j_* = 0.2, and cos *ψ* = −1, resulting in *r_h_* = 1.02062*D* and *U^a^/ϵ* = −0.952 (c) cos *θ_i_* = cos *θ_j_* = 1, resulting in *r_h_* = 3D and *U^a^/ϵ* = −0.965 and (d) cos *θ_i_* = 0.98, cos *θ_j_* = 0.2, and cos *ψ* = 1, resulting in *r_h_* = 2.04156*D* and *U^a^/ϵ* = −0.464.

**FIG. 4.**
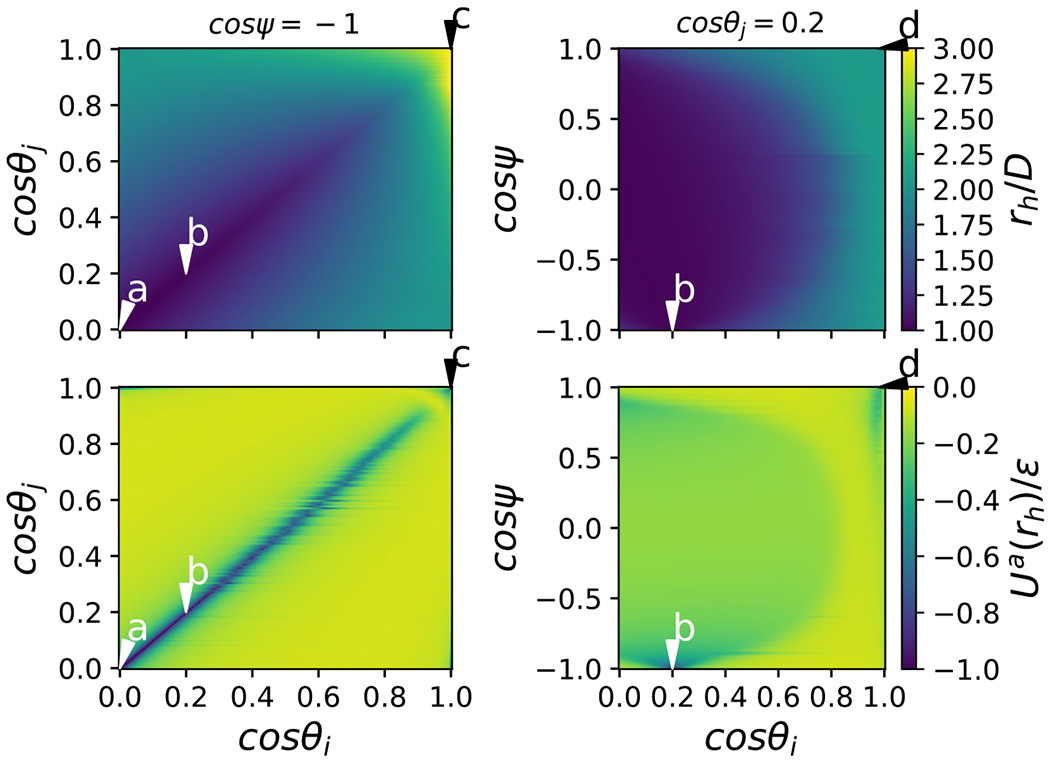
(top) The center separation distance of a pair of cylinders at hard contact, *r_h_*, for *L/D* = 3 and (top left) cos *ψ* = −1, or (top right) cos *θ_j_* = 0.2. Letter-coded labels refer to structures shown in Figure 3. (bottom) The short-range attractive interaction, *U^a^*, at the center separation distance of a pair of cylinders at hard contact, *r_h_*, for *L/D* = 3 and (bottomleft) cos *ψ* = −1, or (bottom right) cos *θ_j_* = 0.2.

**FIG. 5.**
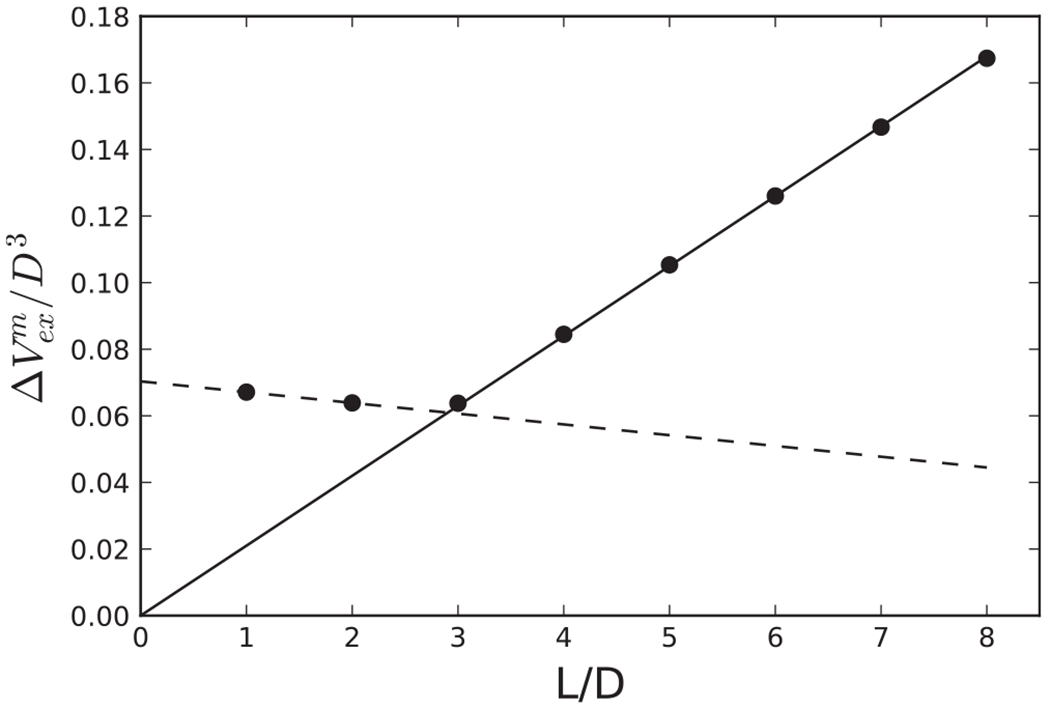
The maximum excluded volume overlap, $\Delta V_{\mathrm{ex}}^{m}$, as a function of the aspect ratio, *L/D*. The solid line is a linear fit for aspect ratios *L/D* ≥ 3, with a resulting slope of 0.021 and zero intercept on the ordinate axis, and the dashed line is a linear fit for aspect ratios *L/D* ≤ 2, which intersects at *L/D* = 2.9.

Examples of the potential energy as a function of center-center distance for four different relative orientations are shown in Figure 2. Additionally, examples of the distance between centers at contact, *r*_*h*_(*θ_i_, θ_j_, ψ*), and the attractive interaction at contact, *U*^*a*^(*r_h_, θ_i_, θ_j_, ψ*), are provided in Figure 4, with sample configurations shown in Figure 3. The configuration where the cylinders are parallel and the axes of symmetry lie in a plane is given by cos *θ*_*i*_ = cos *θ_j_* = 0 and cos *ψ* = 1. As shown in Figure 4 and illustrated in Figure 3(a), this configuration is highly favorable. Also note, in the context of the relative insignificance of Eq. (4) discussed in the previous paragraph, the softness introduced by this term is visually imperceptible and the potential well reaches very nearly a value of −*ϵ* because the contribution of Eq. (4) is relatively small compared to the contribution of Eq. (3). The cylinders may also tilt slightly, as shown in Figure 3(b) and still interact favorably. In addition, because the ends of the cylinders are relatively flat, the attraction of the flat ends, as illustrated in Figure 3(c), is nearly as strong as the parallel configuration shown in Figure 3(a), for an aspect ratio of about *L/D* « 3. As shown in Figure 5, $\Delta V_{\mathrm{ex}}^{m}$ increases linearly with *L/D* for aspect ratios of *L/D* ≥ 3, because the overlap volume in the parallel configuration grows with the length of the cylinder. Note that the slight decrease in $\Delta V_{\mathrm{ex}}^{m}$. shown in Figure 5 for *L/D* ≤ 3 is controlled by *ϵ*_1_ = 0.1. The curvature at the ends of the cylinders gradually increases with length, which leads to a slight decrease in end-end attraction as length increases.

The interaction potential used here requires the calculation of two computationally expensive quantities, *r*_*h*_ and Δ*V_ex_*. The latter is significantly more expensive than the former, and it is not computationally viable to calculate Δ*V_ex_* on-the-fly. It follows that any effort to speed up simulations of these types of systems should focus on speeding up Δ*V_ex_*. Thus, we have adopted to tabulate these interactions as described in Appendix A. Even during the tabulation process, the time to calculate *r*_*h*_ is negligible to that of Δ*V_ex_*. The multi-dimensional tabulation of the anisotropic potential accelerated the simulation by pre-computing these computationally expensive quantities described above over a range of relative positions and orientations, and then interpolating from the stored values during the course of the simulation.

## III. SECOND VIRIAL COEFFICIENT

The second virial coefficient is a useful measure of the relative balance of repulsive and attractions. It is also the central quantity of interest in applying extended corresponding states to compare results obtained from different computational models or experimental measurements. The second virial coefficient, *B*_2_, for solids of revolution is given by^62,63^

(5) $$B_{2}\left(\beta \epsilon \right)=-\frac{1}{4}\int_{0}^{\pi }d\theta_{i}\int_{0}^{\pi }d\theta_{j}\int_{0}^{2\pi }d\psi \int_{0}^{\infty }f\left(r,\theta_{i},\theta_{j},\psi ,\beta \epsilon \right)r^{2}\;\sin\theta_{i}\;\sin\theta_{j}dr,$$

(6) $$f\left(r,\theta_{i},\theta_{j},\psi ,\beta \epsilon \right)=e^{-\beta U\left(r,\theta_{i},\theta_{j},\psi ,\epsilon \right)}-1,$$

where *β* = 1/*k_B_T* and the definitions of the relative orientation variables are described in Section II and Figure 1. In practice, the virial coefficients were computed via Eq. (5) by numerically integrating the tabular potential, as described in Appendix D. The second virial coefficient was split into contributions from the hard particle potential, *U*^h^, and the continuous potential, *U*^*a*^ + *U^e^*, such that $B_{2}=B_{2}^{h}+B_{2}^{\mathrm{ae}}$.

**FIG. 6.**
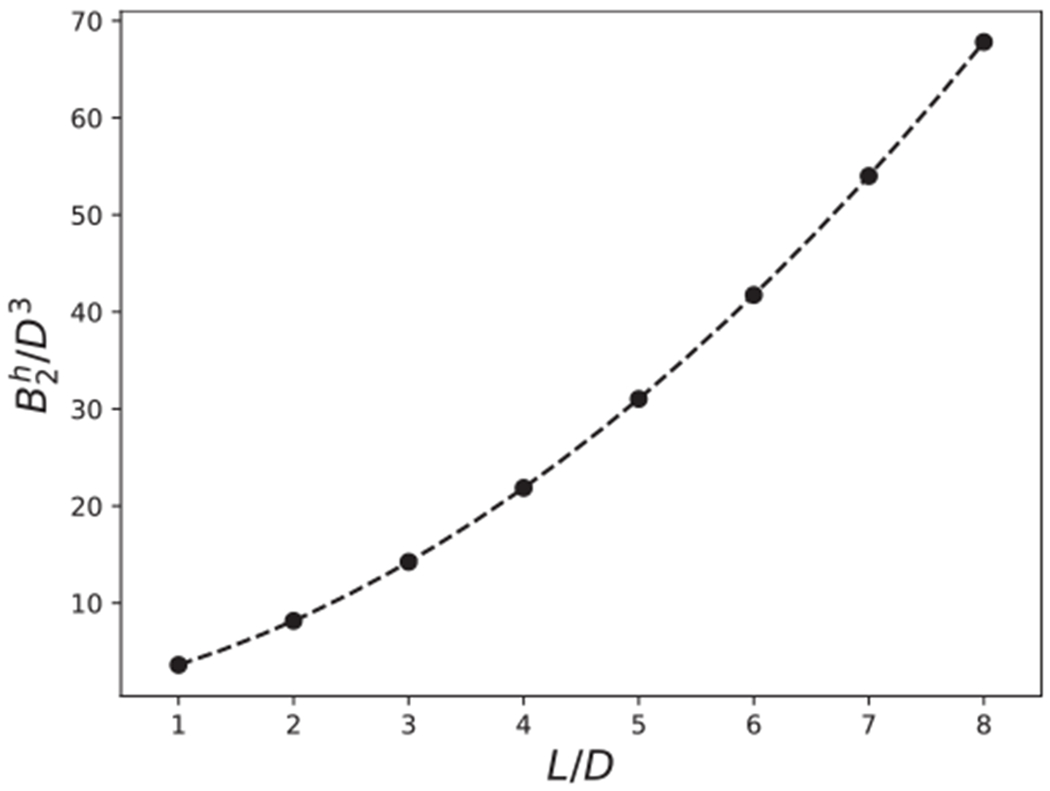
The second virial coefficient, *B*_2_, of hard cylinders (*βϵ* = 0) as a function of the aspect ratio, *L/D*. The line is $B_{2}^{\mathrm{fit}}=0.773+2.22L/D+0.629(L/D{)}^{2}$. The error bars for this Figure, and all remaining figures, show the standard deviation of 3 independent simulations. Error bars are smaller than the size of the symbols and are not visible in this Figure.

**FIG. 7.**
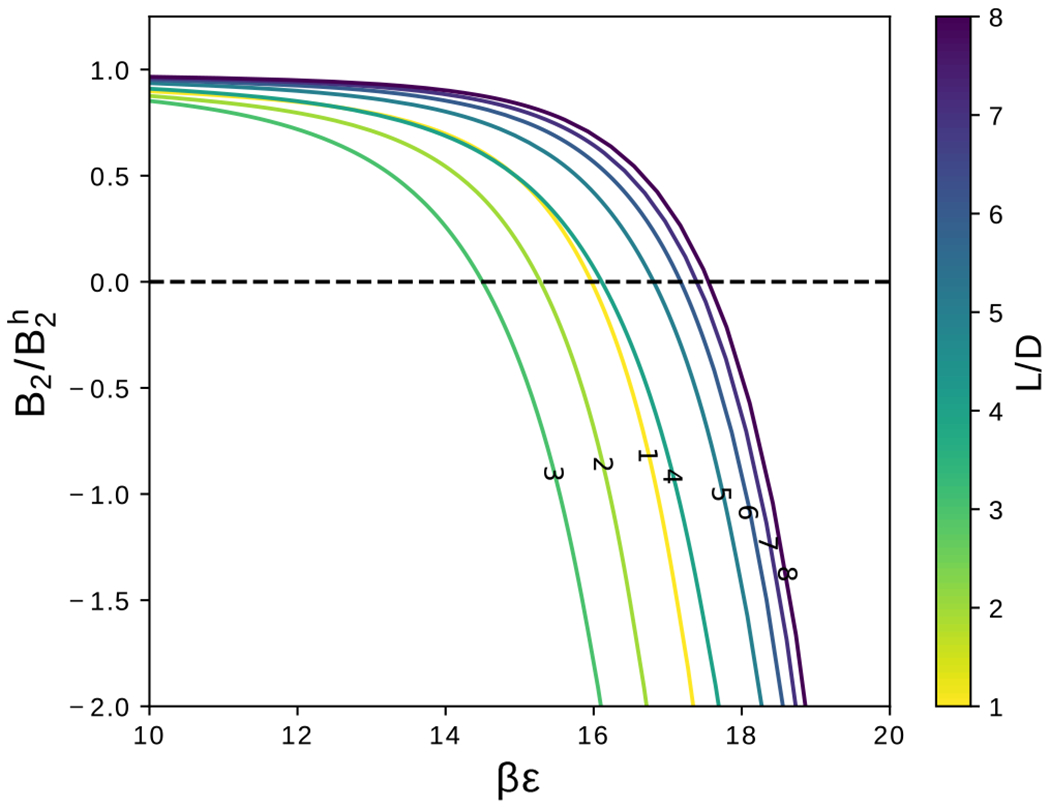
The second virial coefficient, *B*_2_, as a function of the interaction strength, *βϵ*, for integer value aspect ratios in the range *L/D* ∈ [1,8].

The second virial coefficients for hard cylinders with no attractions (i.e., *βϵ* = 0) are shown in Figure 6. These hard-particle virial coefficients are expected to increase with the volume of the particle, here determined by *L*. Comparing with theoretical calculations for hard spherocylinders,^64^ there is expected to be a L^2^ dependence, as appears to be the case in Figure 6. Second virial coefficients for cylinders with various aspect ratios as a function of the interaction strength, *βϵ*, are shown in Figure 7. As the attractive interaction is increased, the virial coefficients become negative. Note that the dependence of *B*_2_ with respect to the aspect ratio follows a similar trend to $\Delta V_{\mathrm{ex}}^{m}$ (see Figure 5).

**FIG. 8.**
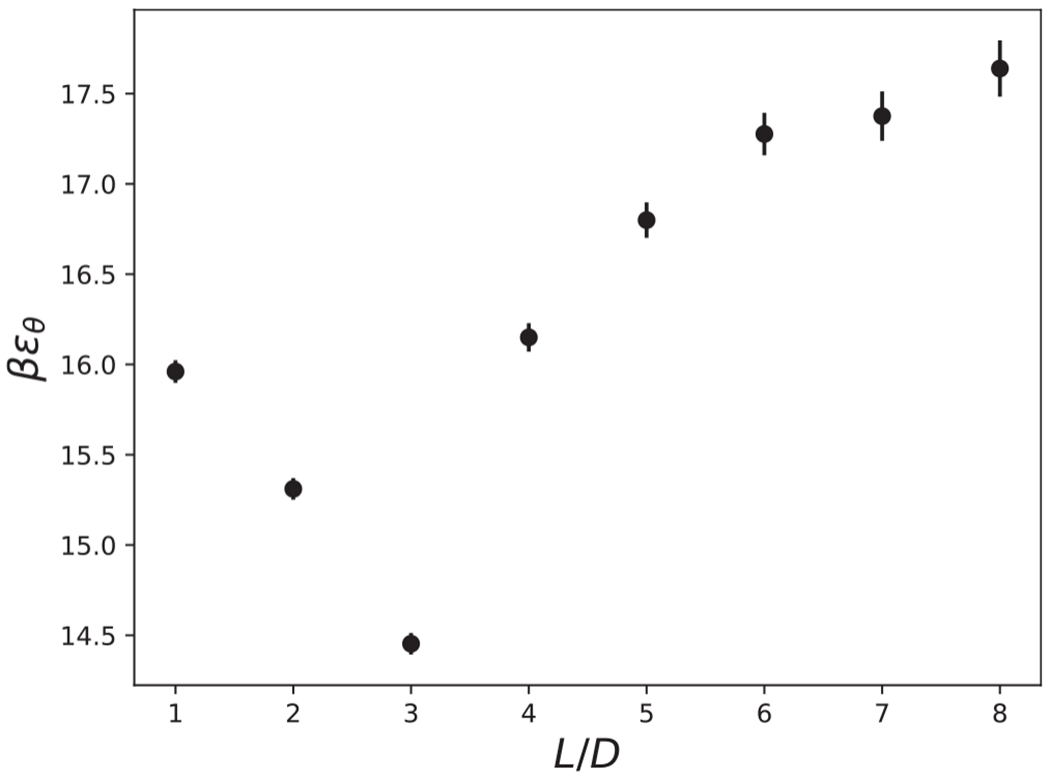
The theta solvent condition, *B*_2_ (*βϵ_θ_*) = 0, is shown as a function of aspect ratio, *L/D*.

The theta solvent condition, *B*_2_(*βϵ_θ_*) = 0, is a convenient parameter to characterize the various shapes, and is shown in Figure 8 and Table I. In practice, the theta solvent condition was obtained by computing *B*_2_ over a large range of values of *βϵ* with a spacing of 5 × 10^−4^. The reported value for *βϵ*_*θ*_ is the average of the following two values of *βϵ*: the maximum *βϵ* for which *B*(*βϵ*) > 0, and the minimum *βϵ* for which *B*(*βϵ*) < 0. The reported error for *βϵ_θ_* is the difference between these two values.

The theta solvent condition, *B*_2_(*βϵ_θ_*_e_) = 0, may be used to identify a cross over from end-end dominant attractions to side-side dominant attractions at approximately *L/D* = 3, as seen in both Figure 5 and 8. For aspect ratios *L/D* < 3, *βϵ*_*θ*_ decreases because the end-end contact interaction dominates energetically, and the end-end contact depends slightly on *L/D*, as discussed previously in Section II for Figure 5. For aspect ratios *L/D* ≥ 3, as the aspect ratio increases, *βϵ*_*θ*_ increases toward an asymptotic value. The location of this cross over depends on both the shape of the end of the cylinder, and also the interaction range, *R*_*g*_. For the remainder of this work, we focus on cylindrical shapes with 3 ≤ *L/D* ≤ 8, because a thorough study of particle shapes with end-end dominant interactions and the effect of end-shape is beyond the scope of this study. In addition, the focus of this work is on aspect ratios of cylindrical particles that were recently synthesized.^43^

## IV. WANG-LANDAU MONTE CARLO SIMULATIONS IN AN EXPANDED ENSEMBLE

Computer simulations employing specialized techniques to sample highly-attractive and short-ranged interactions are used to investigate packing, non-equilibrium gelation and macroscopic phase separation of the cylindrical particles. Wang-Landau (WL) sampling is a flat-histogram method used to obtain the probability distribution function of some specified order parameter. By setting the order parameter to *βϵ*, the expanded ensemble^65,66^ effectively functions as parallel tempering aided by the flat-histogram methods to enhance sampling of transitions with large energy barriers. Note that in this work, the attraction strength, *βϵ*, is trivially related to the temperature, and therefore the expanded ensemble in *βϵ* is analogous to the temperature expanded ensemble. Wang-Landau Monte Carlo with an expanded ensemble in attraction strength enhances sampling of transitions between macroscopic phases and microscopic structural changes.^48^ In addition, specialized Monte Carlo trials are utilized to enhance sampling of clusters which are expected to form due to the strongly attractive interactions. These simulations were conducted using the FEASST simulation package.^67^

The following Monte Carlo trials were employed. Translations and rotations of particles were attempted with equal probability. For expanded ensemble simulations, *βϵ*, was increased or decreased by a fixed amount, ± *δβϵ*, subject to Metropolis acceptance criteria. Collective trial moves were also implemented to facilitate convergence in systems with short-ranged attractions that self-assemble.^46,68^ This included rigid-body translations and rotations of clusters, where clusters were defined as all particles with excluded volume overlap, *r*_*h*_(*θ_i_, θ_j_, ψ*) < *r* < *r*_*c*_(*θ_i_, θ_j_, ψ*), with at least one other particle in the cluster, obtained via a recursive flood-fill algorithm. To obey detailed balance, cluster moves resulting in a particle joining a different cluster were rejected.

The geometric cluster algorithm (GCA) was also used.^46,69,70^ The GCA is a rejection-free algorithm that collectively moves particles, and results in better sampling of clusters of particles than traditional single particle moves. The algorithm proceeds as follows. A particle and a pivot point in space are randomly selected, and the particle is reflected about the pivot. All other particles which interact with the pivoted particle, in both the old and newly pivoted positions, are then attempted to be pivoted with a probability related to the pair interaction energy between the two particles. Each attempted pivot was carried out recursively until all the interacting particles were attempted to be pivoted. To avoid inefficient moves involving most of the particles in the system, the pivot point was confined to a cubic box centered on the first randomly selected particle. The size of this bounding cubic box was tuned during the course of the simulation in order to obtain an average target number of particles involved in a pivot, set to *N*_*p*_/5, where *N*_*p*_ is the maximum number of particles in the simulation. While the rigid cluster moves could not create or destroy clusters due to detailed balance, the GCA does not suffer from this limitation. For anisotropic particles, pivots about a point, as implemented in this work, result in reflections of the particle orientation, and cannot sample all particle orientations without other Monte Carlo trials (e.g., rigid body single-particle and cluster rotations). Note that this is not a deficiency of the GCA method, because particle reflections about a plane and line may be used to sample arbitrary orientations.^71^

The weights for the probability of selecting each trial type are provided in Table II. For each Monte Carlo trial that involved movement of particles, the parameter associated with the maximum change was optimized, via a 5 % change every 10^6^ trials, to yield approximately 25 % acceptance of the trial move.

Simulations were conducted for aspect ratios of *L/D* = [3, 4, 5, 6, 7, 8] and 15 different volume fractions in the interval *ϕ* = [0.05, 0.225], with a spacing of 0.0125. The initial configuration was generated by grand-canonical insertion of *N*_*p*_ particles in a cubic domain of edge length *l*_*b*_/*D* = 20 with periodic boundary conditions in order to obtain a given particle volume fraction, *φ*, rounded to the nearest particle number. Note that volume fractions were computed using particle volumes given in Table I, which were calculated as described in Appendix C. The Wang-Landau update factor, *Inf*, was initially set to unity, and was multiplied by 0.5 whenever the flatness criteria of 80 % was met. See Appendix A of Ref. 72 for implementation details of WL. The simulations were then equilibrated with 2.5 × 10^8^ Monte Carlo trial moves, or 10 update factor reductions (i.e., *Inf* < 10^−6^). Following this equilibration, quantities of interest were averaged, and configurations were stored every 10^6^ trials until at least 14 Wang-Landau flatness conditions were met, although some simulations reached over 37. Each simulation consisted of (3 - 50) × 10^9^ Monte Carlo trials, depending on the conditions, and were run for well over a month of computer time. Expanded ensemble simulations were conducted for a range of *βϵ* in the interval $\left[0.001\Delta V_{\mathrm{ex}}^{m}/(4\pi R_{g}^{3}/3),0.201\Delta V_{\mathrm{ex}}^{m}/(4\pi R_{g}^{3}/3)\right]$, using increments of $\delta \beta \epsilon =0.004\Delta V_{\mathrm{ex}}^{m}/(4\pi R_{g}^{3}/3)$. In order to estimate the standard deviation, all simulations were conducted with 3 identical, independent replicas with different random number seeds. Note that these simulations were well equilibrated and reproducible due in large part to the expanded ensemble flat histogram sampling. Over the course of each simulation, which consisted of 3 - 50 billion Monte Carlo trials, we observed that the system was able to sample fluidly over the range of attraction strengths, indicating that freezing did not occur. Additional simulations were conducted for *l*_*b*_/*D* = 10 and 15 to test the system size dependence of the calculated properties discussed below.

## V. SMALL-ANGLE SCATTERING INTENSITY AND STRUCTURE FACTOR

Small-angle scattering experiments are able to probe short-to-intermediate length scale structures. As such, it is useful to compare the simulations of our model with experiments in order to validate the model and also aid in the interpretation of the experimental results. In order to compare the model and simulations with small-angle X-ray or neutron scattering experiments, as related to the recent experimental synthesis of cylinders and scattering measurements,^43^ the scattering intensity, *I*(*q*), was obtained as the three-dimensional numerical Fourier transform of the scattering density, *ρ*(**r**), averaged over all orientations, Ω,^73,74^

(7) $$I\left(q\right)={\langle {\left|\int_{V}\rho \left(r\right)e^{-iq\cdot r}dr\right|}^{2}\rangle }_{\Omega }.$$

The scattering density was discretized on a grid with 256 elements along each dimension. For each particle, the center point was used to initialize a flood-fill algorithm to fill the spatial grid with scatters if the center point of the grid was inside the particle. The software package FFTW3^75^ was utilized to compute the discrete three-dimensional Fourier transform. Note that discrete Fourier transforms of *ρ*(**r**) naturally take into account the periodicity of the simulation domain. The complex conjugate was then averaged over many uncorrelated configurations stored during the course of the simulations. Orientational averaging of the intensity, *I*(**q**), was performed with channel sharing.^73^ The effective structure factor was obtained by dividing the intensity by the number of particles and by the form factor, which mimics traditional experimental practices. The form factor was computed as the intensity from a single-particle simulation with the same spatial grid resolution.

**FIG. 9.**
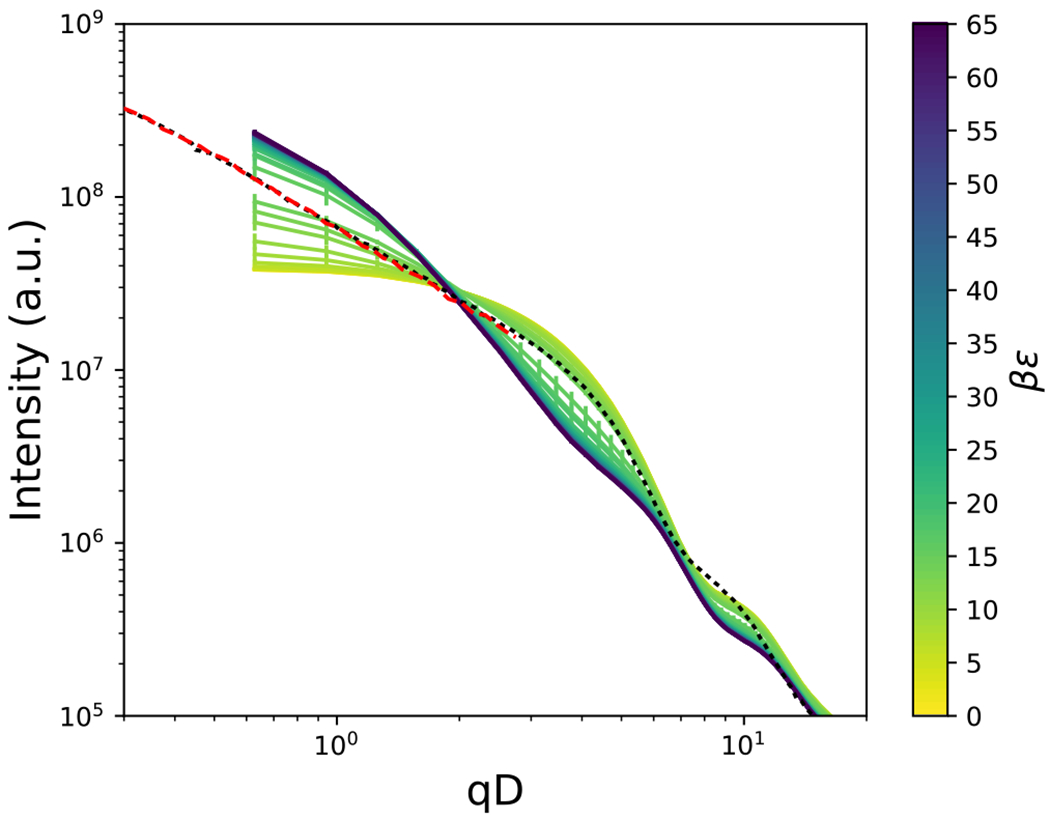
A comparison between scattering intensity from experiment^43,52^ for (black dotted line) USAXS and (red dashed line) USANS at 15 °*C*, *L/D* = 4 and *ϕ* = 0.1125, and simulations shown by the solid lines for varying values of attractive strength, *βϵ*. The experimental scattering intensity was manually shifted by a constant value for comparison.

A comparison between the scattering intensity of experiments and simulations is shown in Figure 9 for an aspect ratio of *L/D* = 4 and a volume fraction of *ϕ* = 0.11 for experiments and *ϕ* = 0.1125 for simulations. To facilitate comparison with experimental scattering intensity results,^43^ smearing functions were utilized to represent the aperture of the instrument and the polydispersity,^76,77^ as described in Appendix E. There is deviation between the experiments and simulations at the lowest *qD* value calculated, but this is expected due to finite size effects of the simulation periodic boundary conditions. In addition, small differences observed for *qD* values above an approximate value of 7 arise from polydispersity of the rods, which were only qualitatively accounted for by smearing the intensity of the monodisperse simulations. In addition to polydispersity, other deviations between experiment and simulation may be due to the approximate modeling of the attractive interactions, which are due to the polymer brush in experiment. This comparison allows us to qualitatively relate the attractive strength, *βϵ*, of the simulations to the temperature in the experiments.

**FIG. 10.**
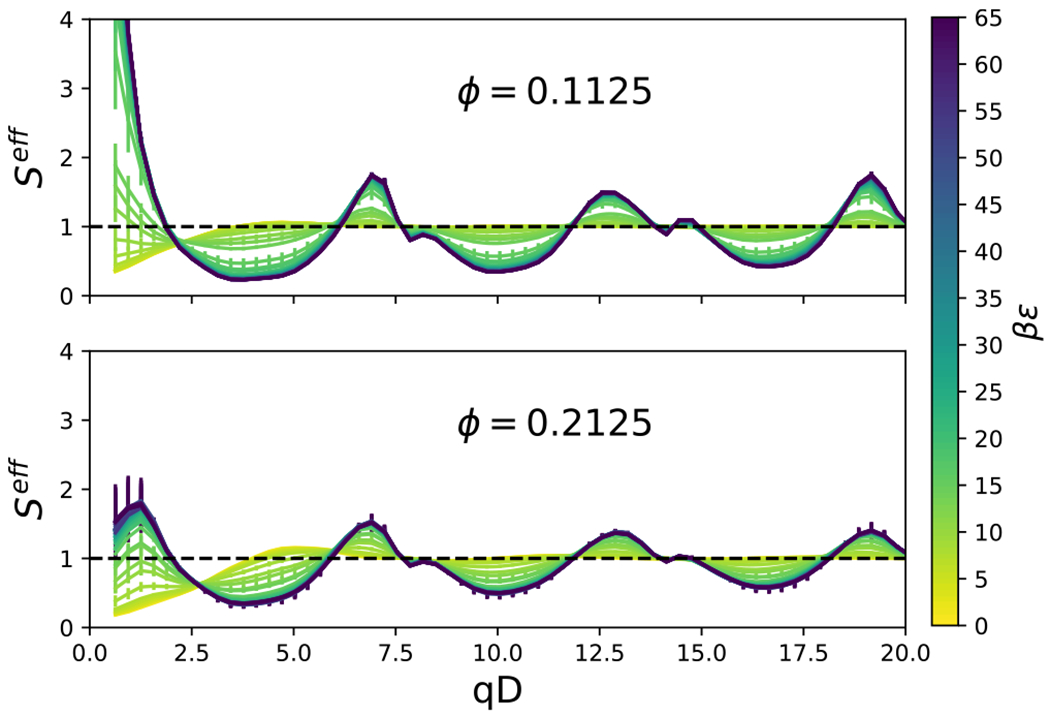
The effective structure factor is shown as a function of *qD* for an aspect ratio, *L/D* = 4, and various attraction strengths, *βϵ*, and volume fractions, *φ*.

**FIG. 11.**
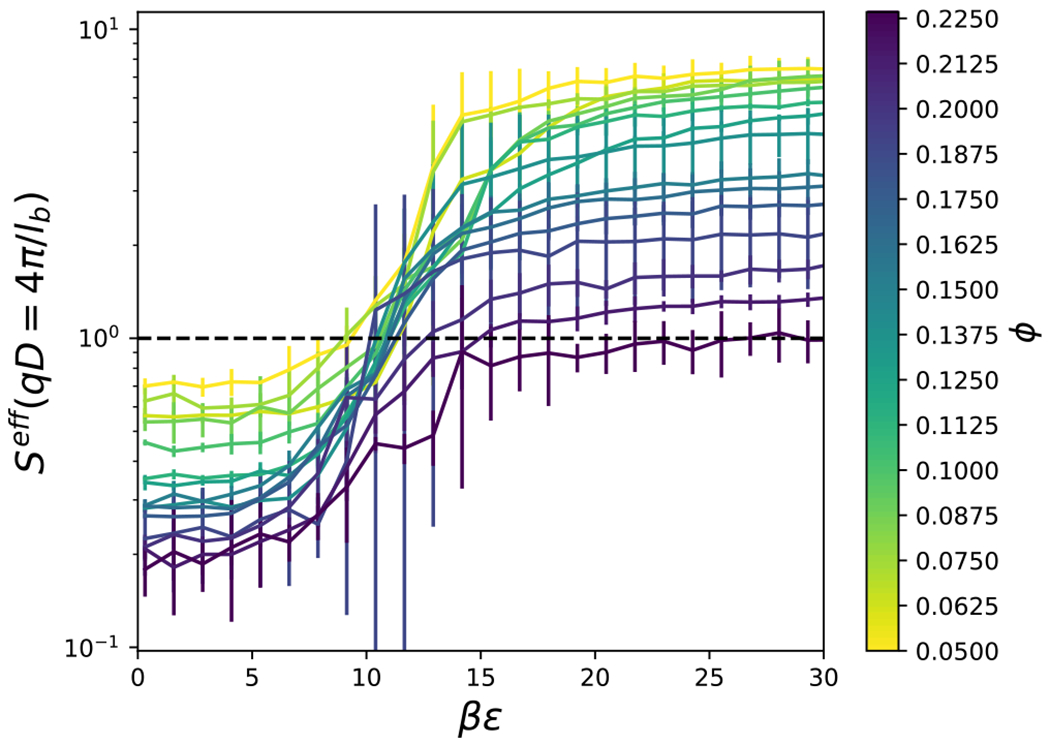
The value of the effective structure factor at the smallest accessible value of *qD* = 4*π/l_b_* is shown for various attraction strengths, *βϵ*, volume fraction, *ϕ*, and *L/D* = 4. In addition, the dashed black line shows where *S^eff^* = 1.

**FIG. 12.**
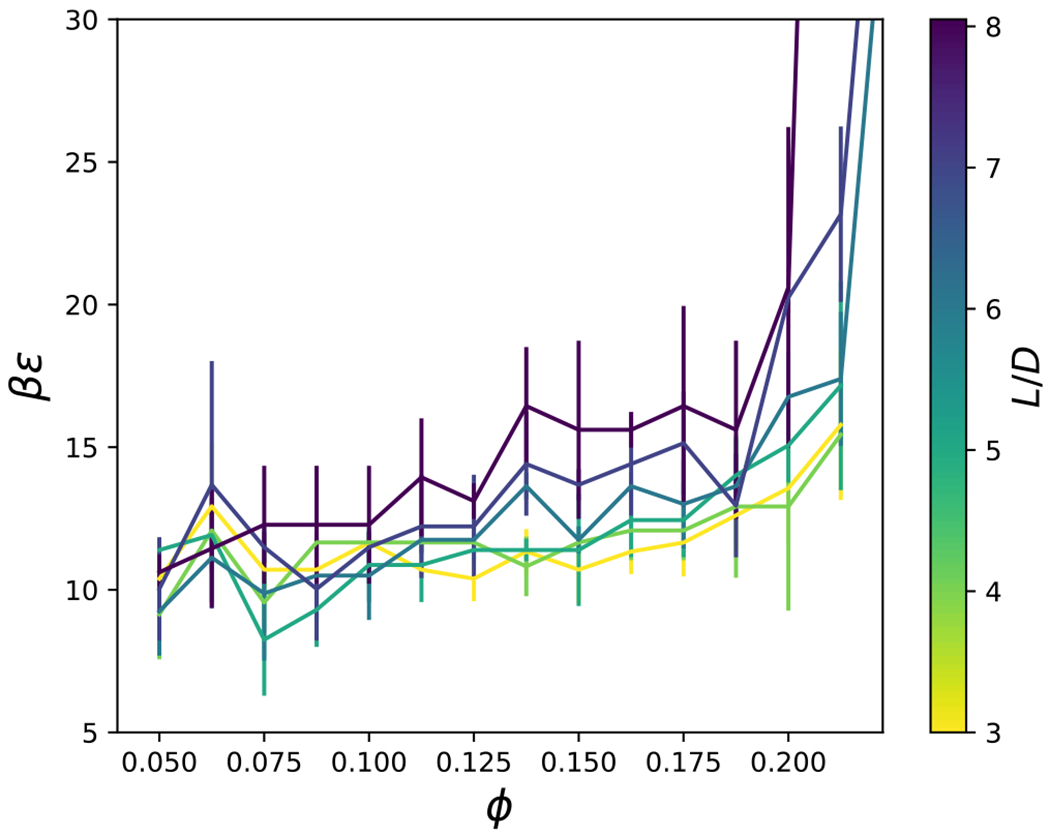
The loci of points where *S^eff^* (*qD* = 4*π/l_b_*) = 1 is shown for a given aspect ratio, *L/D*, attraction strength, *βϵ*, and volume fraction, *ϕ*.

The effective structure factor is shown in Figure 10 for an aspect ratio, *L/D* = 4, and various attraction strengths, *βϵ*, and volume fractions, *ϕ*. As the attractive strength increases, well defined peaks appear in the structure factor, and the low-*qD* values increase. Structure factor values which exceed unity at the low-*q* values (i.e., the largest length scales) are typically associated with macroscopic phase separation, cluster formation or aggregation.^78,79^ Here, the smallest *q* value attainable is *qD* = 4*π/l_b_*, where *l*_*b*_ is the side length of the cubic simulation box. The structure factor for the smallest *qD* values are summarized in Figure 11 for *L/D* = 4. At low attraction strength, *βϵ*, *S*^*eff*^ (4*π/l_b_*) is less than unity, but increases with increasing *βϵ*. The value of *βϵ* at which *S*^*eff*^ (4*π/1_b_*) = 1 tends to increase with increasing packing fraction, *φ*. The increase of *S*^*eff*^ (4*π/1_b_*) upon decreasing density could be an indication that the fluid is metastable with respect to the solid. We do not impose a constraint which forbids the formation of the solid, as previously used in computations with the adhesive hard sphere model.^80^ This is why we do not report on the critical temperature as a function of aspect ratio, as has been studied previously for ellipsoids with an order of magnitude longer range attraction.^81^ As noted previously, our flat-histogram simulations sampled a wide range of attraction strengths without any difficulties, consistent with the avoidance of the stable solid phase. The low-*q S*^*eff*^ results for cylinders with other aspect ratios are summarized in Figure 12, where we plot the attraction strength corresponding to *S*^*eff*^ (4*π/1_b_*) = 1 for a cylinder of given *L/D* and packing fraction, *φ*. An interesting trend that can be gleaned from Figure 12 is that cylinders with smaller aspect ratios tend to exhibit low-*q* structure factors greater than unity more readily than higher aspect ratio cylinders. Analysis of the peaks in the structure factor may be aided by computation of clustering and orientational order, which is presented in the following section.

## VI. CLUSTERS, ORIENTATIONAL ORDER AND PERCOLATION

In the final results section of this manuscript, a detailed analysis of the clustering, percolation and orientational order of the attractive cylinders is presented. In particular, two different measures of clustering will be shown. The first is the average coordination number, *n*_*c*_, which is a local quantity that depends on the nearest neighbors. The second is the probability of connectivity percolation, which is a global quantity that encompasses the entire simulation box, and has been previously shown to be relatively system size independent at the point of 50% probability^40^ and verified for a few of our simulations. In addition, a local and global measure of orientational order will also be shown. The local measure is the angle between axes of revolution of nearest neighbors, and the global measure is the nematic order parameter.

**FIG. 13.**
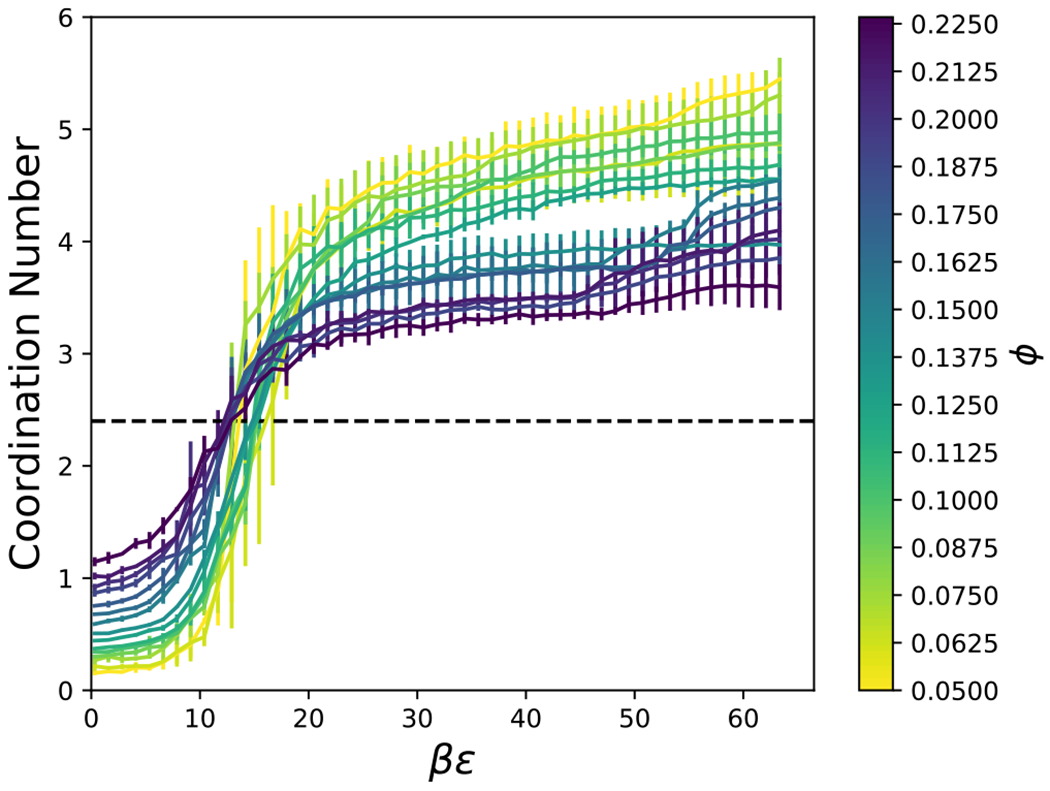
The coordination number for a given interaction strength, *βϵ*, particle volume fraction, *ϕ*, and aspect ratio, *L/D* = 4. The black, dashed line shows a coordination number of 2.4.

**FIG. 14.**
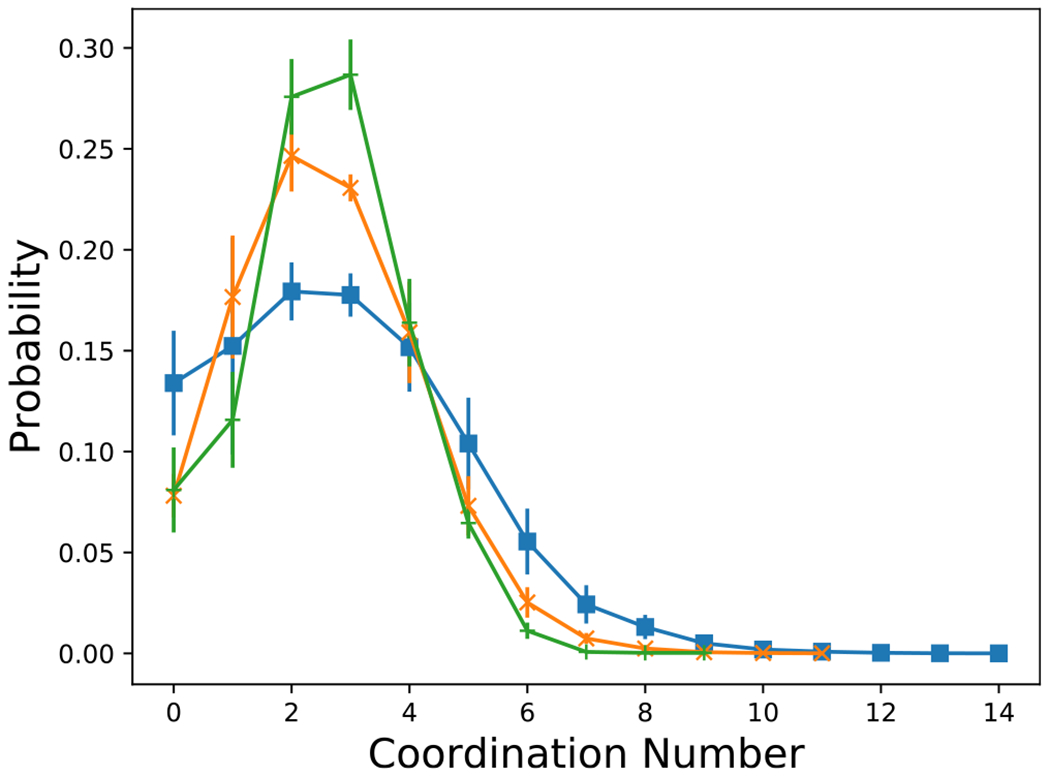
The probability distribution of coordination number, *n_c_*, at the value of *βϵ* nearest an average coordination of 2.4, for (blue square) *L/D* = 4, *ϕ* = 0.1125, *βϵ* = 15.44, where *n_c_* = 2.86 (orange x) *L/D* = 4, *ϕ* = 0.2125, *βϵ* = 12.92, where *n_c_* = 2.6 and (green +) *L/D* = 8, *ϕ* = 0.1125, *βϵ* = 20.6, where *n_c_* = 2.58.

**FIG. 15.**
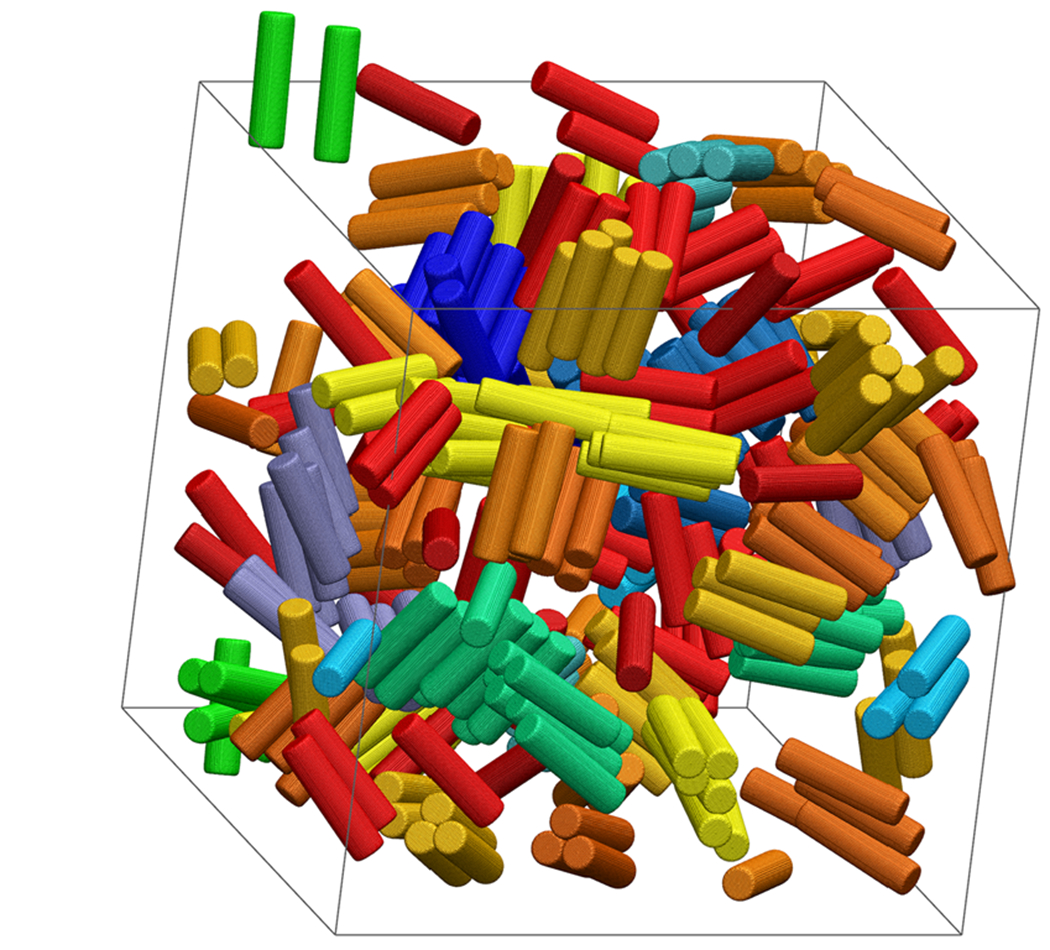
Cylinders colored on a red-green-blue gradient according to the number of cylinders in a cluster with *L/D* = 4, ϕ = 0.1125 and *βϵ* = 15.44. The average coordination number is 2.4. The periodic boundaries are shown in gray.

**FIG. 16.**
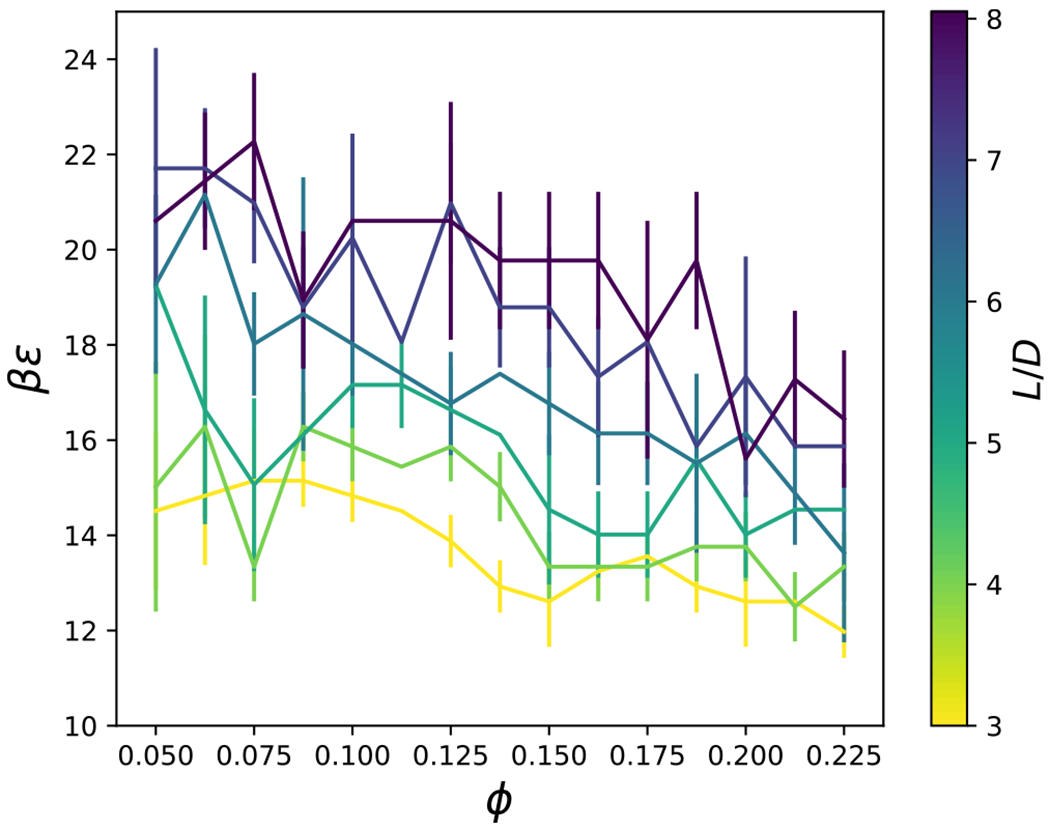
The loci of points where the coordination number is 2.4, for a given interaction strength, *βϵ*, particle volume fraction, ϕ, and aspect ratio, *L/D*.

Rigidity percolation has been proposed to occur at 〈*n_c_*〉 = 2.4,^82^ which has been shown previously to indicate gelation and dynamical arrest in isotropic model systems.^39,42^ The coordination number, *n*_*c*_, was computed as the average number of particles, denoted by *i*, whose exclusion volume overlaps with a central common particle, denoted by *j* ≠ *i* (e.g., when *r*_*h*_(*θ_i_, θ_j_, ψ*) < *r* < *r*_*c*_(*θ_i_, θ_j_, ψ*)). As shown in Figure 13 for *L/D* = 4, the coordination number increases with increasing attractive strength, *βϵ*, as expected. For low attraction strengths, when *n*_*c*_ ≲ 2.4, the coordination number tends to increase with volume fraction; however, this trend reverses when *n*_*c*_ ≳ 3. Figure 14 shows the distributions of *n*_*c*_ at conditions where the average, 〈*n*_*c*_〉 ≈ 2.4. Because the overall shape of these distributions resemble those of previously published spherical particles,^39^ the underlying physics of rigidity percolation in the different systems could be similar. However, a more detailed comparison with the distributions in the literature is complicated by the difficulty in finding the conditions where *n*_*c*_ = 2.4, precisely. An example configuration of the cylinders with 〈*n*_*c*_〉 = 2.4 is shown in Figure 15 for *L/D* = 4, *ϕ* = 0.1125 and *βϵ* = 15.44. Figure 16 shows that the attraction strength, *βϵ*, at which 〈*n*_*c*_〉 = 2.4, increases with increasing aspect ratio, *L/D*. In addition, the attractive strength corresponding to 〈*n*_*c*_〉 = 2.4 tends to decrease with increasing volume fraction. Also notice that a coordination number of 2.4 can be found at all packing fractions, even when the packing fraction is too low to allow percolation. This is due to the formation of clusters. The coordination number criterion for rigidity percolation should be used with some caution for freely diffusing particle systems.

**FIG. 17.**
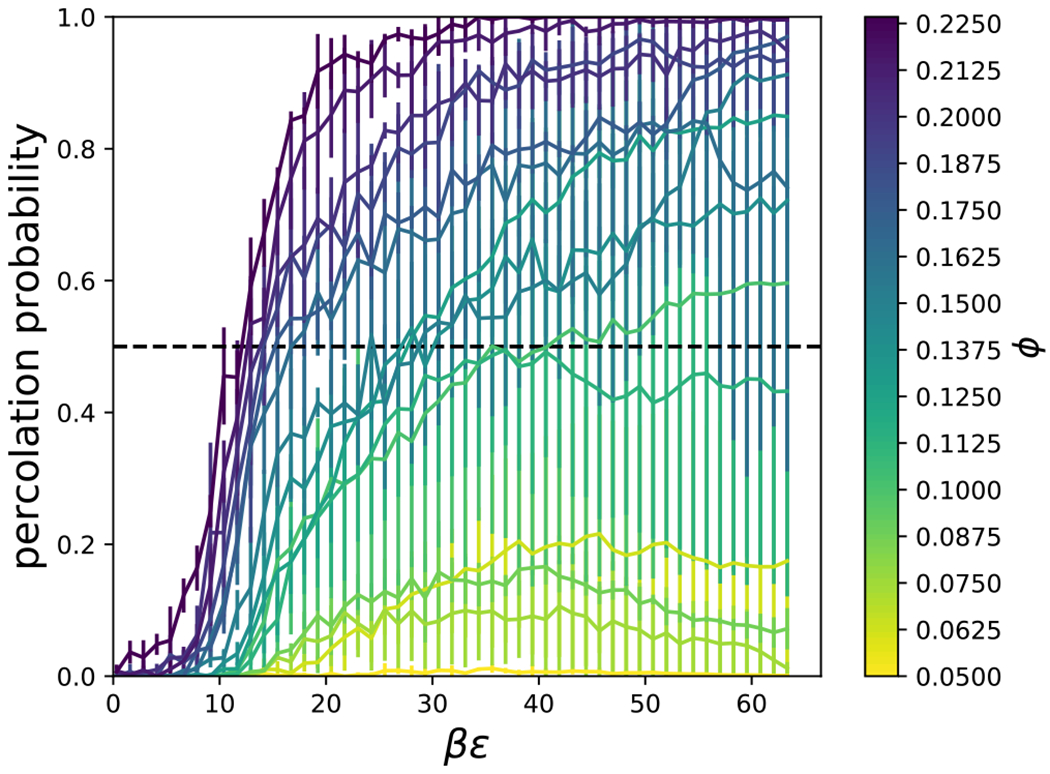
The probability that there is a percolated cluster for a given interaction strength, *βϵ*, particle volume fraction, *ϕ*, and aspect ratio, *L/D* = 4. The black, dashed line shows the 50 % probability.

**FIG. 18.**
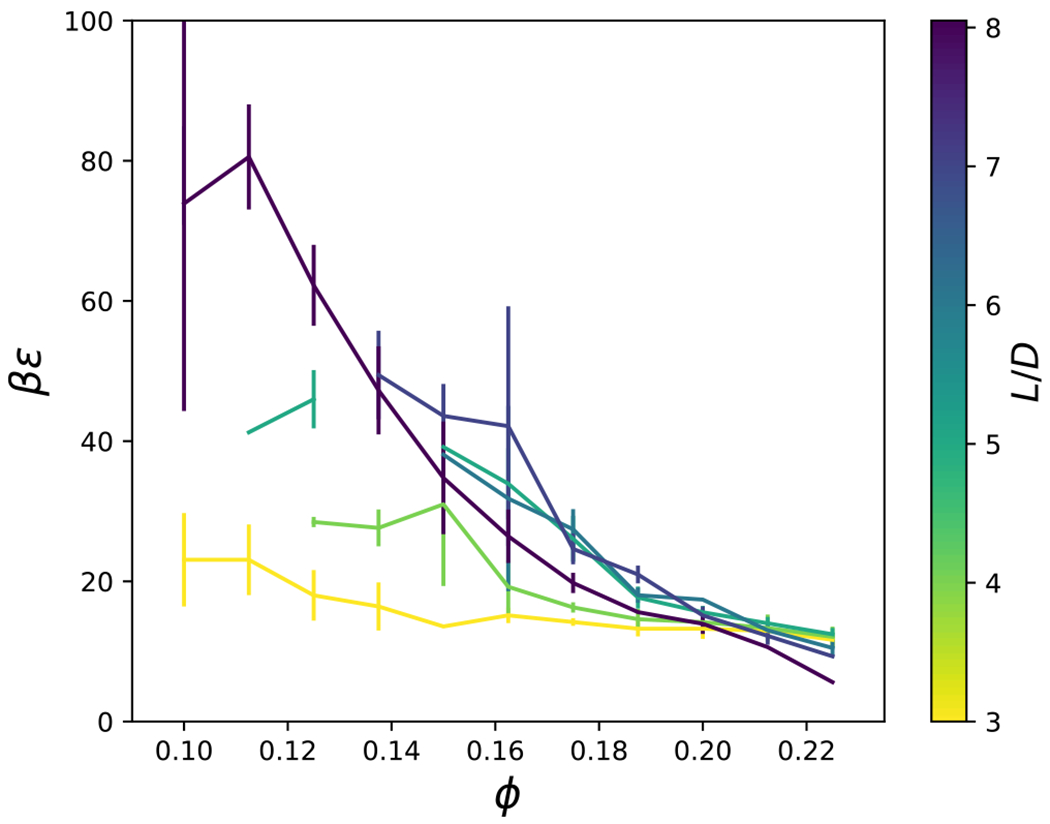
The loci of points when there is a 50 % probability that a cluster is percolated, for a given interaction strength, *βϵ*, particle volume fraction, *ϕ*, and aspect ratio, *L/D*.

Percolation of clusters also serves as an important metric for relating structural quantities to dynamical arrest. Clusters of particles were defined as all particles with overlap of their excluded volumes, *r*_*h*_(*θ_i_, θ_j_, ψ*) < *r* < *r*_*c*_(*θ_i_, θ_j_, ψ*), with at least one other particle in the cluster, obtained via a recursive flood-fill algorithm. A cluster was considered percolated if the particles in the cluster were connected to their own periodic images.^40^ The percolation of clusters of cylindrical particles was investigated as a function of the interaction strength, *βϵ*, the particle volume fraction, *φ*, and aspect ratio, *L/D*. In Figure 17, we plot the percolation probability for *L/D* = 4. As the volume fraction, *ϕ*, and interaction strength, *βϵ*, increase, the probability of percolation increases. The point at which there is a 50 % probability of percolation was shown to be system size independent in Ref. 40 and verified for a few of our simulations (not shown). Figure 18 shows that the attraction strength, *βϵ*, corresponding to 50 % percolation probability generally increases with aspect ratio, and decreases with packing fraction. There is a minimum packing fraction below which percolation does not occur.

**FIG. 19.**
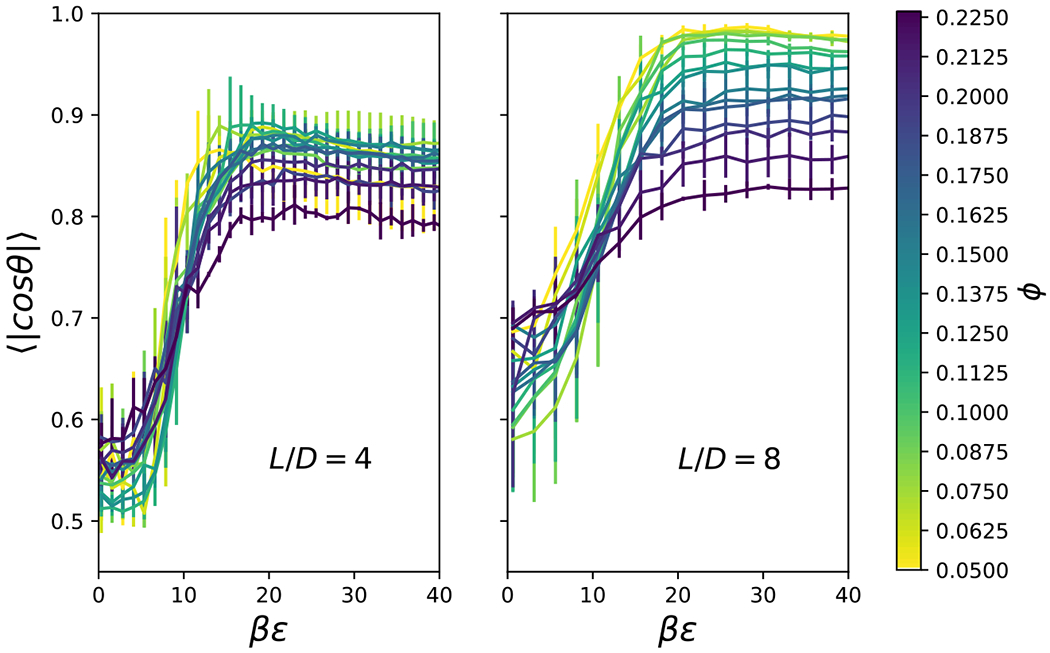
The ensemble average of the absolute value of the cosine of the angle between axes of revolution of rods with surfaces within a distance 0.08*D* as a function of interaction strength, *βϵ*, particle volume fraction, *ϕ*, and aspect ratio, *L/D*.

The orientation of the rods was investigated with two different order parameters. One measure is the angle between the axes of revolution, denoted by *θ*, between neighboring particles with excluded volume overlap, *r*_*h*_(*θ_i_, θ_j_, ψ*) < *r* < *r*_*c*_(*θ_i_, θ_j_, ψ*). Thus, this is a measure of local orientational order. The absolute value of the cosine of this angle, | cos *θ*|, is unity for parallel configurations, and the ensemble average of this quantity, 〈| cos *θ*|〉, has an average expectation value of 0.5 for random orientation.^74^ As shown in Figure 19, the local orientational order increases with attractive strength, *βϵ*, and slightly decreases with packing fraction, *φ*. This trend for 〈| cos *θ*|〉 is very similar to that observed for the coordination number in Figure 13, because similar physical mechanisms dictate the behavior of both of these local ordering measures. For *L/D* = 4, 〈| cos *θ*|〉 does not reach unity even for high attractive strengths, *βϵ*. Although the parallel orientation is strongly favored, a perpendicularly-oriented cylinder may interact favorably with a cluster of parallel cylinders which present an approximately flat face.

**FIG. 20.**
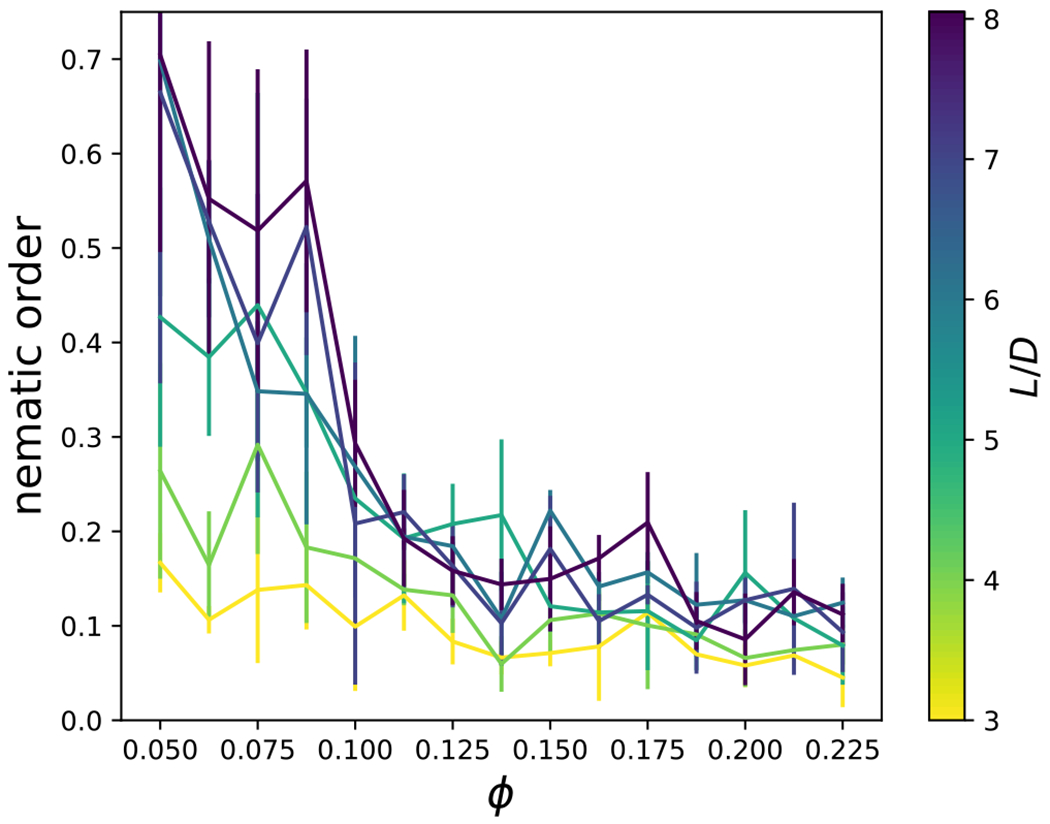
The nematic order parameter is shown at the highest attractive interaction strength investigated as a function of particle volume fraction, *ϕ*, and aspect ratio, *L/D*.

In addition, the nematic order parameter was computed as the largest eigenvalue of the tensor **Q** = (2*N*)^−1^ Σ^*N*^(3**u**_*i*_**u**_*i*_−*I*).^40^ The nematic order parameter is unity when rods are fully aligned, and zero in a perfectly isotropic fluid. Figure 20 shows that the nematic order parameter rarely reaches a value above 0.5. Nematic order decreases with packing fraction because the glassy and gel-like configurations are less likely to sample the states with global orientational order. Furthermore, for larger aspect ratios, particles are more likely to orient globally.

## VII. CONCLUSIONS

The structural properties of cylindrical particles with short-range attractions were investigated using Wang-Landau Monte Carlo computer simulations over a wide range of attraction strengths, volume fractions and aspect ratios. The second virial coefficients were calculated for different aspect ratios and attraction strengths. Interestingly, *B*_22_ exhibits a crossover from end-end dominant attractions to side-side dominant attractions as a function of *L*/*D*. The scattering intensity compared well with experiment, and the low wave-number effective structure factor provides a useful measure for the structure of the cylinders. The conditions where percolating clusters form were investigated with two independent measures: connectivity of the clusters across periodic boundaries and also 2.4 coordination number, which has been related to rigidity percolation.^82^ The orientations of the rods were also investigated with two different measures. First, the relative orientation of the axes of symmetry of neighboring particles, which is a local measure of orientational order, was reported. Then, the nematic order parameter was also shown, and this is a global measure of orientational order. These measures suggest the rods may possess orientational order at length scales corresponding to nearest-neighbors, but the global orientational order of the entire system rarely, if ever, reaches a full nematic phase. Instead, small crystallites with local order pack together into more disordered configurations.

This manuscript lays the groundwork for more detailed comparisons with experimental results of the gelation line in the temperature-packing fraction projection of the phase diagram obtained from recently synthesized cylindrical particles.^43,44^ In particular, a method has been developed to collapse this gelation line with respect to aspect ratio.^52^ This method involves fitting the structure factor to a model for an adhesive hard sphere in order to quantify the temperature of gelation for general potentials and models.

Other future work includes a more thorough analysis of particles with *L/D* < 3, where end-end interactions become dominant. For example, one interesting phenomena observed at *L/D* = 1 was that the particles stacked into larger cylinders which resembled the rouleaux of red blood cells.^83^ Finally, the methodology for efficiently simulating particles according to the superquadric equation will be used to investigate the phase behavior of a variety of different anisotropic-shaped particles in future studies.

## Figures and Tables

**TABLE I.** Properties of cylinders as a function of the aspect ratio.

*L/D*	*V_p_/D*^3^	$\Delta V_{\mathrm{ex}}^{m}/D^{3}$	*βϵ_θ_*
1	0.779585	0.06712	15.96(6)
2	1.55918	0.06388	15.31(6)
3	2.33877	0.06378	14.45(6)
4	3.11837	0.08448	16.15(8)
5	3.89797	0.10535	16.8(1)
6	4.67758	0.12603	17.3(1)
7	5.45717	0.14671	17.4(1)
8	6.23672	0.16739	17.6(2)

**TABLE II.** Monte Carlo trials and relative weights for the probability of selection.

trial	weight
single-particle translation or rotation	5
cluster translation or rotation	1/5*N_p_*
geometric cluster algorithm	1/*N_p_*
*βϵ* change	1/100

**FIG. 21.**
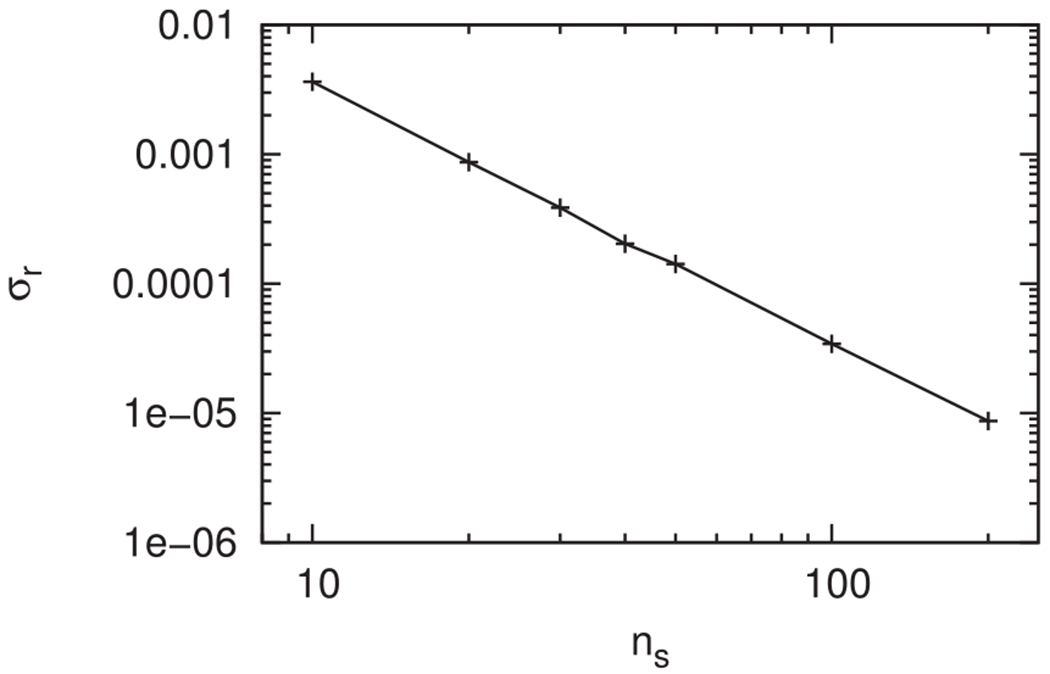
Convergence of the number of surface points for a sphere, *a_x_* = *a_y_* = *a_z_* = 0.5 and *ϵ*_1_ = *ϵ*_2_ = 1 averaged over 57 046 orientations.

**FIG. 22.**
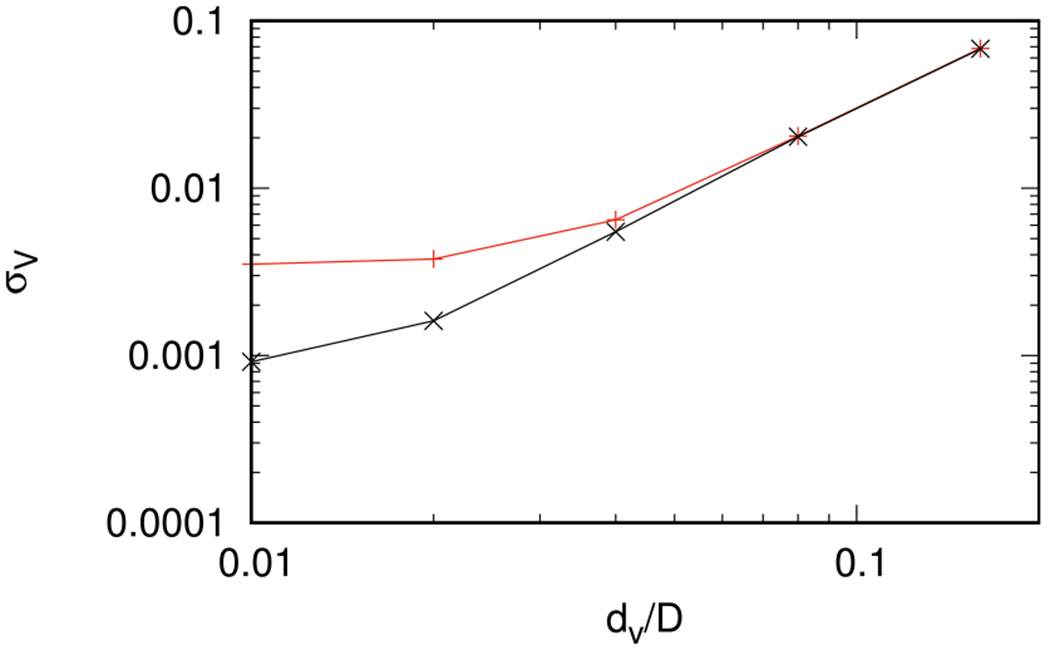
Convergence of the voxel side length, *d_v_* with *n_t_* = 128 for a sphere, *a_x_* = *a_y_* = *a_z_* = 0.54 and *ϵ*_1_ = *ϵ*_2_ = 1 for (red +) *n_s_* = 50 and (black x) *n_s_* = 100.

**FIG. 23.**
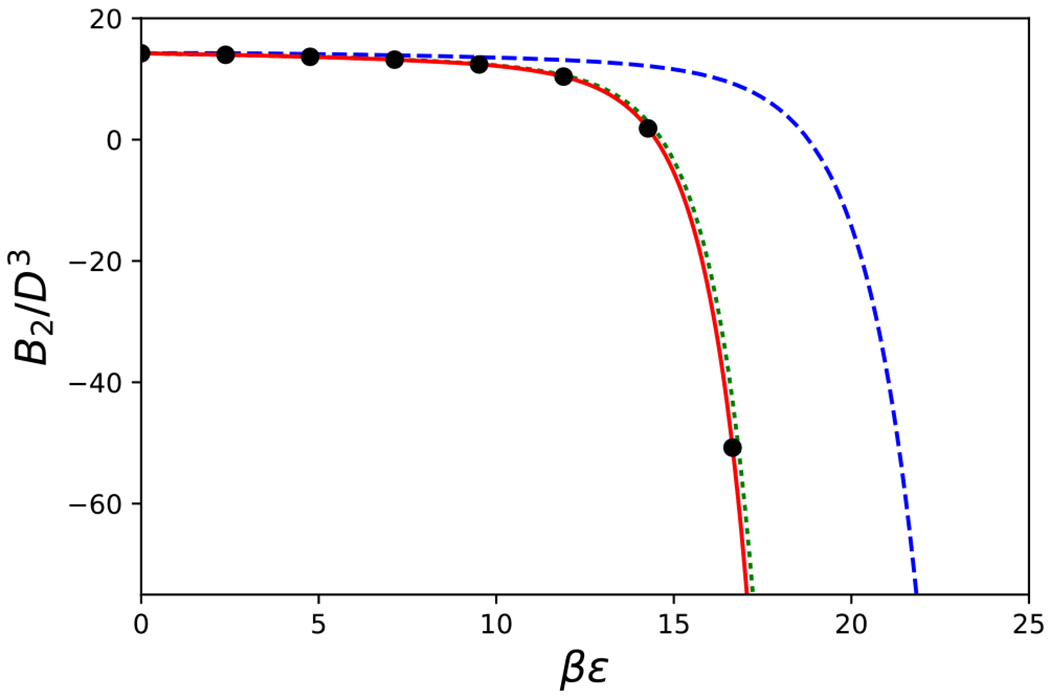
The second virial coefficient of a cylinder with *L/D* = 3 as a function of interaction strength, *βϵ*, for the following values of *n_ez_*: (dashed blue line) 1, (dotted green line) 10, (solid red line) 100 and (black points) 1000.

## APPENDIX A: TABULAR POTENTIAL

The tabular potential was constructed to obtain, *r_h_*(*θ_i_, θ_j_, ψ*), *r_c_*(*θ_i_, θ_j_, ψ*) and *U^a^*(*r, θ_i_, θ_j_, ψ*) efficiently during the course of the simulation, as described in Section II, where *r_c_* is the center separation distance at which the edges of the excluded volumes of the particles touch. The construction of the tabular potential is similar to that described in the Appendix of Ref. 48 for two dimensional particles. In this work, the algorithm to find the contact distance, as described in Appendix B, also returned the surface point which was closest to the contact. This surface point was subsequently used to obtain the initial voxel of the recursive flood-fill algorithm to compute the overlap volume, as described in Appendix C, over the range of *n_z_* + 1 values of $z=\frac{r-r_{h}}{r_{c}-r_{h}}$, *z* ∈ [0, 1].

In this work, the tabulated angles were evenly spaced over cos *θ_i_*, cos *θ_j_* and cos *ψ*, in order to avoid the arccos operation during the simulation. Because the shapes are symmetric about the plane which possesses the center point and is perpendicular to the axis of symmetry, negative values of cos *θ* did not need to be tabulated, and cos *θ* ∈ [0, 1] over a range of *k* + 1 evenly-spaced values. No symmetry operation on *ψ* was necessary because cos *ψ* ∈ [−1, 1] over a range of 2*k* + 1 evenly-spaced values. Nearly half of the possible combinations of cos *θ_i_* and cos *θ_j_* may be ignored by swapping *i* and *j* if cos *θ_i_* > cos *θ_j_*, which is also required for unique computation of the potential energy due to the randomness in Monte Carlo integration. In this work, *k* = 150, which was shown to be sufficient in Ref. 48 and resulted in 6 863 101 tabulated orientations. As a measure of the expected error in the tabular potential due to Monte Carlo integration, two tables with identical parameters, and *L/D* = 3, but different random number seeds were compared. The square root of the sum of the squared difference of *U^a^*(*r^h^*)/*ϵ* was 4 × 10^−5^, and these squared differences did not vary greatly with orientation. During the course of the simulations, values of *r_h_, r_c_* and *U^a^* are obtained for arbitrary orientation by multi-dimensional linear interpolation. Example two-dimensional slices of the tabular potential are shown in Figure 4. In addition, with *n_z_* = 4, the tabular potential stores in less than 1 gigabyte of memory. This smaller value of *n_z_* was used to capture the qualitative shape of the implicit-depletant potential, but more precision was not necessary when one intends to use extended corresponding states to compare with experiments, by matching second virial coefficients between models and experiments.

## APPENDIX B: HARD CONTACT

The following algorithm used to obtain the distance between centers at hard contact is less efficient than alternative algorithms.^54,55^ But the efficiency of this algorithm is not an issue for two reasons. First, this algorithm is not utilized during the simulations because the tabular potential described in Appendix A requires these contact distances to be computed only once before the simulations begin. Second, the bottleneck in the interaction between the particles is the attractions, and not the hard contact.

The distance between the centers at hard contact is obtained by decorating the surface of the particles with many points, and checking whether those surface points overlap with the other particle. For a given relative orientation, *r_h_*(*θ_i_, θ_j_, ψ*) is obtained by incrementally moving a particle center by an amount *δ* until the overlap status changes, and then halving *δ* and changing its sign, until *δ* < 10^−15^. Surface points are obtained by solving the implicit equation, Equation (1) in evenly-spaced grids for two of the three independent variables for the following three cases: the x-y plane, the x-z plane, and the y-z plane. Each of these grids was of size (*n_s_* + 1, *n_s_* + 1) and spanned the range of the respective size parameter “*a*” for the relevant dimension. In addition, the parametric equations with longitude and latitude parameters and a similar-sized grid, was also used to obtain a fourth set of surface points. Combining these four cases ensures that there are no large gaps in coverage of the surface. In this work, *n_s_* = 100, which resulted in 64 838 points for the cylinders, and 57 046 for a sphere, because the edges of the Cartesian planes may have no solution for Equation (1).

The number of surface points was tested by the convergence of the contact function of two spheres of unit diameter (*a_x_* = *a_y_* = *a_z_* = *D*/2 and *ϵ*_1_ = *ϵ*_2_ = 1) for 57 046 orientations. Because each sphere was represented by a mesh of surface points, each orientation may deviate from the theoretical center separation distance of unity, and these deviations diminish as the number of surface points increase, as shown in Appendix D. The square of the deviations for each orientation was summed and plotted as a function of *n_s_*. Convergence was assessed by the quantity $\sigma_{r}=\sqrt{\sum_{n}^{N=57046}(1/N)(r_{h}(i)-1{)}^{2}}$, which is the standard deviation from the theoretical result. As shown in Figure 21, the contact function calculation with *n_s_* = 100 represents a sphere within a standard deviation of approximately 3.4*D* × 10^−5^.

## APPENDIX C: OVERLAP VOLUME

The following algorithm was used to compute the overlap of the excluded volumes of two particles during the tabulation of the potential before the simulations were run, as described in Appendix A. Because this algorithm is not used during the course of the simulations, the efficiency of the algorithm is not the simulation bottleneck as long as the tabular potentials do not take too long to compute for a given pair of shapes. Here we used a combination of a recursive flood-fill algorithm and Monte Carlo integration. In this algorithm, space is divided into a grid of voxels with side length of *d_v_*. To begin, a voxel is selected which is known to contain an overlap. Determining this voxel is described more in the following Appendix A on construction of the tabular potential. This selected voxel, and all neighbors of this selected voxel are then subject to Monte Carlo integration with *n_t_* random test points. A test point is inside a shape described by Equation (1) if the “inside outside” function, which is the left hand side of Equation (1), is less than or equal to unity. For each test point, if the point is inside both shapes, then there is overlap at that point. The volume of overlap within the voxel is determined by the number of test points which are inside the overlap. For each voxel that is computed by Monte Carlo integration and found to have overlaps, all neighbors of that voxel are then considered for Monte Carlo integration recursively. This algorithm applies to pairs of concave shapes which are ensured to have a single contiguous region of overlap.

The parameters for the overlap volume are now compared with theory for the spherical case. The accuracy of the overlap volume is determined by the average density of test points, here given by $n_{t}/d_{v}^{3}$. The number of test points in a voxel, *n_t_*, is chosen to be the smallest value with no appreciable overhead cost for the flood-fill algorithm. For example, if *n_t_* is too small (e.g., *n_t_* = 1) then the algorithm slows down, for a given average point density, due to increased memory to store the voxels for the flood-fill algorithm. But if *n_t_* is too large, for fixed point density, then the flood fill algorithm is not fully utilized. The side length of the voxel, *d_v_*, was tested against the overlap of two contacting spheres with diameter of 1.08 and a center separation of 1 averaged over 57 046 realizations of random numbers. Theoretically, the overlap volume is known to be given by *V_o_* = *π*(2*R* − *d*)^2^ (*d* + 4*R*)/12 for two equal-sized spheres of radius *R* = 0.54*D* and separation distance *d* = *D*. Thus, convergence may be assessed by the quantity $\sigma_{V}=\sqrt{\sum_{i}^{N=57046}(1/N)((V(i)-V_{o})/V_{o}{)}^{2}}$, which may be thought of as the percent standard deviation. As shown in Figure 22, *σ_V_* reaches a plateau that depends on the number of surface points, *n_s_*. This plateau occurs when the relative precision of the contact function due to *n_s_* becomes higher than the precision due to *d_v_*. For the simulations of the cylinders, *d_v_* = 0.05D and *n_t_* = 512, which has a similar point density as *d_v_* = 0.03*D* and *n_t_* = 128 for the sphere case, where overlap volumes were computed within an average precision lower than 0.01 percent.

The volume of a single particle, *V_p_*, was computed by the same algorithm as described here, with *n_t_* = 10^5^ and *d_v_* = 0.06*D*. The resulting values of *V_p_*, reported in Table I were utilized to determine volume fractions. Note that, with *ϵ*_1_ = 0.1, the volumes of the cylinders are within 0.74 % of the expected cylindrical volume of $V^{p}/D^{3}\frac{L}{D}\frac{\pi }{4}$.

## APPENDIX D: COMPUTATION AND CONVERGENCE OF THE SECOND VIRIAL COEFFICIENT

The tabulated interaction described in Appendix A can be used directly to calculate the second virial coefficient, *B*_2_, using Equation (5). Note that the tabulated potential applies to solids of revolution possessing an axis of rotational symmetry and a plane of symmetry at the center point and perpendicular to the axis of symmetry. In other words, we use the following

(D1) $$\int_{0}^{\pi }f\;\sin\;\theta d\theta =2\int_{0}^{\pi /2}f\;\sin\;\theta d\theta ,$$

(D2) $$\int_{0}^{2\pi }fd\psi =2\int_{0}^{\pi }fd\psi .$$

The tabular potential described in Appendix A may be conveniently integrated according to this equation with some changes of variables. In particular,

(D3) $$\int_{0}^{\pi /2}f\;\sin\;\theta d\theta =\int_{0}^{1}fd\left(\cos\;\theta \right),$$

(D4) $$\int_{r_{h}}^{r_{c}}f\left(r,\ldots \right)r^{2}dr=\int_{0}^{1}f\left(z,\ldots \right)r^{2}\left(r_{c}-r_{h}\right)dz,$$

where $z=\frac{r-r_{h}}{r_{c}-r_{h}}$ and *r_c_* is the center separation ”cut off” distance at which the edges of the excluded volumes of the particles touch. Note that no change of variable for *ψ* was used to avoid numerical instabilities due to a singularity. In addition, the second virial coefficient was split into contributions from the hard particle potential, *U^h^*, and the continuous potential, *U^a^* + *U^e^*, such that $B_{2}=B_{2}^{h}+B_{2}^{\mathrm{ae}}$. For the hard particle contribution,

(D5) $$\int_{0}^{\infty }f^{h}\left(r,\theta_{i},\theta_{j},\psi ,\beta \epsilon \right)r^{2}dr=-\frac{1}{3}r_{h}{\left(\theta_{i},\theta_{j},\psi \right)}^{3},$$

where *f^h^* = *e*^−*θU^h^*^ − 1. Thus, the second virial coefficient contribution from the hard particle potential, *B^h^*, is obtained by substitution of Equations (6), (7), (D1)–(D3), and (D5) into Equation (5),

(D6) $$B_{2}^{h}=\frac{2}{3}\int_{0}^{1}d\left(\cos\;\theta_{i}\right)\int_{0}^{1}d\left(\cos\;\theta_{j}\right)\int_{0}^{\pi }r_{h}{\left(\theta_{i},\theta_{j},\psi \right)}^{3}d\psi .$$

The second virial coefficient contribution from the continuous interaction, $B_{2}^{\mathrm{ae}}$, is obtained by substitution of Equations (6), (7), (D1)–(D4) into Equation (5),

(D7) $$B_{2}^{ae}\left(\beta \epsilon \right)=-2\int_{0}^{1}d\left(\cos\;\theta_{i}\right)\int_{0}^{1}d\left(\cos\;\theta_{j}\right)\int_{0}^{\pi }d\psi \int_{0}^{1}f\left(z,\theta_{i},\theta_{j},\psi ,\beta \epsilon \right)r^{2}\left(r_{c}-r_{h}\right)dz.$$

The numerical integration was then performed using a multi-dimensional trapezoidal rule, which is expected to converge well for periodic functions. Because *z* is tabulated with lower resolution than *θ* and *ψ*, the resolution of *z* used to perform the integration was increased by a factor of 100 with respect to the resolution of the tabular potential. To show that *n_ez_* = 100 is sufficient, the second virial coefficient was computed for a number of interpolated values in *z, n_ez_*, as shown in Figure 23. Direct integration of the table is expected to be more efficient than Monte Carlo integration (e.g. Ref. 48) because a very small portion of configuration space contributes significantly, especially at relatively high values of *βε* and *L/D*.

## APPENDIX E: SMEARING OF THE SCATTERING INTENSITY

For slit aperture smearing,

(E1) $$I_{s}\left(q_{i}\right)\approx \overset{N-1}{\underset{j=1}{\sum }}\left[\sqrt{q_{j+1}^{2}-q_{i}^{2}}-\sqrt{q_{j}^{2}-q_{i}^{2}}\right]I\left(q_{i}\right)$$

(E2) $$\approx \overset{N-1}{\underset{j=i}{\sum }}W_{ij}I\left(q_{j}\right)$$

where *W_ij_* = 0 when *q*_*j*+1_ − *q_i_* > Δ*q* = 0.117Å^−1^. See the SASVIEW 4.1.2 manual^77^ for derivation of the above equation in the smearing functions section. Using a diameter of 2610Å for comparison to the experimental system,^43^ Δ*qD* = 305.37.

For polydispersity smearing, we assume that the diameter of the cylinder follows a Gaussian function and that the scattering intensity scales with the diameter of the cylinder. This assumes that the length and the diameter are correlated and follow the same distribution and that the polydispersity has no effect on the relative packing of all the cylinders. Following these assumptions, the smeared intensity is given by

(E3) $$I_{s}\left(q_{i}\right)=\int_{0}^{\infty }\frac{1}{\sqrt{2\pi \sigma_{q}^{2}}}\exp\left[-\frac{{\left(D_{i}-D_{j}\right)}^{2}}{2\sigma_{q}^{2}}\right]I\left(q_{j}\right)dD_{j},$$

where *q_j_D_j_* = *q_i_D*. In order to implement this polydispersity smearing on the simulation data, the integration variable is changed from *D_j_* to *q_j_* and converted to a summation. In addition, the Gaussian width parameter, *σ_q_*, was truncated at 3*σ_q_*. Finally, the Gaussian width parameter was normalized such that *σ_q_* = Δ*_q_D_j_*, where Δ_*q*_ = 0.135. Note that we normalize by *D_j_* instead of *D* so that the discretized Gaussian kernel width does not depend on *q_j_*. The working equation is given by

(E4) $$I_{s}\left(q_{i}\right)\approx \overset{N-1}{\underset{j=i}{\sum }}\exp\left[-\frac{{\left(q_{i}-q_{j}\right)}^{2}}{2{\left(q_{i}\Delta_{q}\right)}^{2}}\right]\frac{q_{i}}{q_{j}}I\left(q_{j}\right).$$

In order to apply the smearing kernel at the minimum and maximum *q* values, *I*(*q_j_*) was extended assuming it was constant.
